# Ultrasound-guided dry needling versus traditional dry needling for patients with knee osteoarthritis: A double-blind randomized controlled trial

**DOI:** 10.1371/journal.pone.0274990

**Published:** 2022-09-30

**Authors:** Johnson C. Y. Pang, Amy S. N. Fu, Stanley K. H. Lam, B. Peng, Allan C. L. Fu

**Affiliations:** 1 School of Health Sciences, Caritas Institute of Higher Education, Hong Kong, China; 2 Department of Rehabilitation Sciences, The Hong Kong Polytechnic University, Hong Kong, China; 3 The Hong Kong Institute of Musculoskeletal Medicine, Hong Kong, China; 4 Department of Family Medicine, The Chinese University of Hong Kong, Hong Kong, China; 5 Department of Family Medicine, The University of Hong Kong, Hong Kong, China; 6 Department of Rehabilitation Medicine, Sichuan Provincial People’s Hospital, Chinese Academy of Sciences Sichuan Translational Medicine Research Hospital, University of Electronic Science and Technology of China, Chengdu, Sichuan, China; 7 Discipline of Physiotherapy, Sydney School of Health Sciences, The University of Sydney, Sydney, Australia; 8 Musculoskeletal Research Hub, Charles Perkins Centre, The University of Sydney, Sydney, Australia; Monash University, AUSTRALIA

## Abstract

**Objective:**

To compare the effect of ultrasound (US)-guided dry needling (DN) with traditional DN in the treatment of pain and dysfunction for patients with knee osteoarthritis (KOA).

**Design:**

A double-blind, randomized controlled trial.

**Methods:**

Patients (25 male and 65 female), age 50–80 years diagnosed with KOA were recruited and randomly assigned to one of three groups in a 1:1:1 ratio for intervention: real US-guided DN with exercise therapy (G1), placebo US-guided DN with exercise therapy (G2), and exercise therapy solely (G3). G1 and G2 were blinded to the application of real or placebo US guidance by turning the monitor of US imaging out-of-view from participants’ vantage points. The effectiveness of blinding was evaluated by asking the participants whether they had received real-US guided DN. The responses were assessed by Chi-square test. Visual Analogue Scale (VAS), Knee injury, and Osteoarthritis Outcome Score (KOOS) subscales (KOOS-pain, KOOS-symptoms, KOOS-quality-of-life (QoL)) were collected at baseline, 4 weeks, and 8 weeks by a blinded assessor. Data were analyzed by mixed model analysis of variance (ANOVA) with Bonferroni correction.

**Results:**

Eighty-four participants (61.26±5.57 years) completed the study. G1 achieved significant improvement in VAS at 8 weeks compared to G2 and G3 (G1 vs. G2: MD = -15.61, 95% CI [-25.49, -5.51], *p* = 0.001; G1 vs. G3: MD = -19.90, 95% CI [-29.71, -10.08], *p*< 0.001). G1 achieved significant improvement in KOOS-pain at 8 weeks compared to G2 and G3 (G1 vs. G2: MD = 9.76, 95% CI [2.38, 17.14], *p* = 0.006; G1 vs. G3: MD = 9.48, 95% CI [2.31, 16.66], *p* = 0.010). KOOS-symptoms and KOOS-QoL were not statistically significant between groups. G2 had no significant difference of the perceptions as G1 with p = 0.128. G2 were successfully blinded to placebo US-guided DN.

**Conclusion:**

US-guided DN with exercise therapy may be more effective than traditional DN with exercise therapy or exercise therapy alone in reduce pain of KOA.

## Introduction

Knee osteoarthritis (KOA) is the most common type of osteoarthritis in the elderly community [1] and its prevalence is rising as the population ages [2]. The prevalence of KOA is projected to increase from 26.6% to 29.5% by 2032 of the proportion of the population aged ≥ 45 years [3]. KOA accounts for approximately 80% of today’s total medical expenditure on osteoarthritis [4].

KOA is a degenerative disease characterized by a progressive articular cartilage deterioration, resulting in pain and severe disability. However, KOA’s etiology is unclear [5]. Among the medical interventions, total knee replacement (TKR) is commonly employed for the management of KOA [6]. An estimated 3.48 million total knee replacements will be performed by the year of 2030 in USA [7]. However, approximately 30% of patients after TKR have experienced chronic pain postoperatively [8, 9] and minimal effects on quality-of-life (QoL) [10]. Apart from surgical interventions, the long-term use of pharmacological interventions may also unavoidably increase the risk of medical complications [11]. To reduce the medical burdens, and complications of surgical and pharmacological interventions, the use of non-pharmacological intervention has been recommended by the European League Against Rheumatism (EULAR) [12], the American College of Rheumatology (2012) [13], and the World Health Organization (WHO) (2019) [14].

Among the non-pharmacological interventions, dry needling (DN) is one of the modalities used to treat KOA by physiotherapists that has been found cost-effective and superior to acupuncture [15]. Timely non-pharmacological intervention may be the key to delay disease progression [16]. DN, traditionally, is a puncturing method involving the insertion of needles into tender spots of the human body without the injection of any substance. In the treatment of knee pain, favorable outcomes may result from the release of tight soft tissue around the tibiofemoral joint and/or patellofemoral joint, specifically the medial patellofemoral ligament, medial patellotibial ligament, medial collateral ligament, patella tendon, and joint articular retinaculum [17–20]. Microtrauma triggered by the puncturing of soft tissue through DN can elicit inflammatory responses that activate mast cells proliferation [21], release anti-inflammatory cytokine IL-10 [22], and promote soft tissue healing [23]. The repeated puncturing through the US-guided DN can mechanically disrupt the scar tissue and induce bleeding that may drive growth factor–β and basic fibroblast growth factor [24, 25]. In addition, DN piercing of the soft tissue may improve blood circulation, decrease peripheral and central sensitization, and release the neurotransmitters serotonin and noradrenaline [26, 27].

DN’s effectiveness is dependent on both the stimulation intensity and the accuracy of identifying the affected anatomical structures for needle insertions [28]. Through traditional DN has demonstrated favorable outcomes in some studies of patients with shoulder pain [29], KOA [30–35] and hip osteoarthritis [36, 37], others have not been able to induce significant improvements [38–40]. These inconsistencies may be traced to the absence of a standardized approach and inaccuracies related to needle targeting and advancement.

In contrast, US-guided DN has shown favorable outcomes in some painful musculoskeletal conditions, such as shoulder pain, myofascial pain syndrome [28, 40], chronic neck pain [41], carpal tunnel syndrome [42], and tendinopathy [43–45]. The pairing of US-guidance with DN assists in identifying the precise location of anatomical structures and advancing the needle to the intended tissues [20]. US is a low-cost and effective imaging technique that could guide percutaneous procedures without the risk of ionizing radiation [46].

To our knowledge, our RCT is the first to compare the effectiveness of US-guided DN versus traditional DN therapy in the management of pain and dysfunction in patients with KOA.

## Materials and methods

### Study design

We conducted a double-blind randomized controlled trial (RCT) in adherence to Consolidated Standards of Reporting Trials (CONSORT) statement and in accordance with the Helsinki Declaration. Ethical approvals were obtained from both the Human Subjects Ethics Sub-committee of The Hong Kong Polytechnic University and the Research and Ethics Committee of the Caritas Institute of Higher Education (CIHE). The study received approval from the WHO International Clinical Trials Registry Platform (Reference number: ChiCTR2000033581). The protocol can be accessed in the following: https://www.protocols.io/view/title-ultrasound-guided-dry-needling-versus-tradit-cgh5tt86 [DOI: dx.doi.org/10.17504/protocols.io.3byl4j2zjlo5/v1]

### Participants

Ninety participants (25 male and 65 female) age 50 to 80 years (61.26±5.57) with KOA were recruited from public by paper and digital advertisements. Their diagnoses of KOA were confirmed by an orthopedic surgeon. Their x-rays reports were read by a registered radiologist for the classification of KOA. Then, a registered physiotherapist screened participant’s eligibility based on the inclusion and exclusion criteria as stated below. Participants were provided with participant information sheets, and verbal explanations. Written consents were provided, and participants could withdraw from the study at any time. To protect the participants’ privacy, data were collected and concealed in a password-protected computer file only accessible by the principal investigator in the physiotherapy laboratory of CIHE. Adverse effects, if any, were recorded and reported in this study. The recruitment was stopped when adequate participants (ninety) were achieved.

### Inclusion and exclusion criteria

Inclusion criteria were based on previous relevant studies by using needling techniques for KOA management [34, 47]. Inclusion criteria were as follows: 1) >50 to 80 years old, 2) referred with a diagnosis of KOA (i.e., primary KOA fulfilling the American College of Rheumatology criteria developed by Altman et al. (1986) [48], (at least three out of six) for clinical and radiographical diagnostic KOA 3) presenting with anterior and/or medial knee pain with 2–3 local tender spots, 4) crepitus, 5) functional limitations caused by KOA over a period of at least six months, 6) stiffness <30 minutes, 7) KL scale [49] of grade I to grade III; and 8) able to read Chinese and communicate in Cantonese based on literacy.

Exclusion criteria, made with reference to previous studies [34, 47], were: 1) having knee pain less than 6 months; 2) having other musculoskeletal diseases associated with knee pain (e.g., referred pain from the low back or posterior or lateral knee pain or co-existing pain over the other limb); 2) suffering from acute inflammation, diffuse tenderness upon palpation test, bone marrow lesion, severe joint deformity with X-rays revealing a grade IV in the KL scale [49], coagulation disorders, metabolic, or neuropathic arthropathies, immunosuppressed or systematic disease; 3) having severe concomitant illness that might affect the clinical outcomes of this study, 4) contraindications to DN including pregnancy, malignancy, fear of DN; 5) having previous experience of treatment involving acupuncture or DN therapy, or recent non-pharmacological intervention (e.g., physiotherapy) within a month prior to the start of the study; 6) inability to answer questionnaires and non-responsiveness towards the assessor; 7) having a wound or pressure sore or skin problems or skin allergy, including an allergy to iodine; and 8) having a history of injecting steroids.

### Randomization and blinding

Eligible participants’ baseline data was obtained and randomly assigned into one of three groups in a 1:1:1 ratio for a 4-week intervention by an independent research assistant, who did not participate in the data collection or interpretation of the results, using a randomization software. The sequence of group allocation was concealed until interventions were assigned by the independent research assistant with sealed and stapled opaque envelopes. Group one (G1) received real US-guided DN with exercise therapy; Group two (G2) received placebo US-guided DN with exercise therapy; and Group three (G3) received exercise therapy solely. To blind the participants in G1 and G2, the monitor of ultrasound guidance for DN was turned off in G2 and out-of-view from both G1 and G2 participants’ vantage points. The effectiveness of blinding was evaluated by asking the participants whether they had received a real-US guided DN. Additionally, outcomes were assessed at baseline, after treatments at 4 weeks and 8 weeks by an independent assessor who was blinded to the allocation or intervention. Therefore, both participants and assessor were blinded in this study to reduce the risk of performance and detection biases.

### Interventions

#### Experimental groups

G1 participants received treatment provided by a registered physiotherapist specialized in US imaging and DN intervention. The participant was positioned in a supine position on a bed with the affected knee joint supported by a towel at 30° flexion. An US machine with high frequency (4-12MHz) linear probe (Laboratories ANIOS US probe and system, Model: L12-4 broadband linear array; Philips Lumify) was used to assess any patello-femoral and medial patello-tibial compartments [18] with attention paid toward the following: mucoid degenerative changes (**Fig 2A**), heterogeneity (**Fig 2B and 2C**), or the presence of thickening of ligaments, osteophytes, meniscus tear or hyaline cartilage thinning in trochlear cartilage defects, aligning with the standards set by the Outcome Measures in Rheumatology Ultrasound Task Force on KOA [18, 50]. To avoid anisotropy and confirm pathology, both long- and short-axis views were taken [18]. The whole procedure of US scanning, findings of the heterogenicity and mucoid degenerative changes under the sonographic examination were analyzed and recorded by the physiotherapist in **S1 File**.

Corroboration of the US imaging results with a detailed physical examination to select the sites of needling. The DN with US-guided was then performed for the pathological structures (**Fig 3**) using sterile stainless needles (Pipe handle; Dong Bang Acupuncture, INC, Gatineau, Canada) with the size of 0.3 x 40 mm [51]. The procedure was performed by the physiotherapist with sterile technique by disinfecting the US probe with a wet towel (Clinell Universal Wipes, GAMA Healthcare Ltd, Hemel Hempstead, Hertfordshire), covered it with a tegaderm (3M; St. Paul, MN, USA), and used Aquasonic sterile transmission gel (Aquasonic sterile transmission gel; Parker Laboratories, INC) for the transmission of US energy to the participants. The physiotherapist also disinfected the participants’ skins with antiseptic solution (Betadine antiseptic solution; Mundipharma Pharmaceuticals LTD, Dail, Cyprus) over the targeted DN sites.

The DN technique, guided by that documented in previous studies [52, 53], involved slowly moving the needle in-and-out of the muscle or tendon with anticipation of an appropriate response, identifiable as a local twitch response (LTR), a dull aching or sense of heaviness or distension, or reproduction of the participant’s symptoms. The needle was then manipulated in-and-out of the targeted tissue five times every five minutes over a fifteen-minute span. After the treatment, sterile gauze (A.R. Medicom Inc. (Asia) Ltd, HKSAR, China) was applied and pressed on the DN site. Sterile non-stick pads (Orison TM, HKSAR, China) were used to cover the sites that received DN. The number of needles applied was dependent on the participants’ condition; the maximum used on a single patient was seven [54]. The participants in G2 experienced a similar protocol with the exception that the procedure incorporated a placebo, rather than a real, US-guidance technique. The same physiotherapist who had performed the procedure for G1 patients simulated the act of scanning while the US monitor’s screen, out-of-view from the G2 patient’s vantage point, was turned off to blind the participants.

In addition, all participants received a routine exercise program as reported by using TIDieR guidelines (Details of the exercise program in: http://www.tidierguide.org/#/gen/dHUwKw-7u), and education of knee care by a video once per week for four weeks. The content of the educational video included basic anatomy, pathology, daily live activities modification to avoid overloading of knee joint, etc. The exercise therapy was conducted under the supervision of a registered physiotherapist weekly for 4 sessions. The participants were instructed to continue the exercises at home three times per week for another four weeks.

#### Control group

G3 participants received only routine exercise program and educational materials related to the care of their knees identical to those of G1 and G2. The exercise therapy was also conducted under the supervision of a registered physiotherapist weekly for 4 sessions and instructed to continue the exercises at home three times per week for another four weeks as per G1 and G2. The exercises focused on improving knee mobility, soft tissue flexibility, muscle strength, balance and proprioception.

### Outcome measures

#### Primary outcomes

*Subjective pain intensity*. The Visual Analogue Scale (VAS) was measured with a 100 mm horizontal line anchored with labels on top of it [55, 56]. This method is superior to numeric pain rating scale in measuring KOA pain with good reliability. The minimum clinically important difference (MCID) was 19.9 mm [57, 58]. The participants reported the average of current pain level at that moment.

*Disability measures*. The Knee injury and Osteoarthritis Outcome Score (KOOS)-short form, a reliable and validated tool, was used to measure the level of disability for study participants.

#### Secondary outcomes

*Compliance of exercise*. Exercise compliance for participants was asessed at 4 weeks and 8 weeks by an independent assessor. The duration of exercise was asessed by hour per week.

*Medication utilization during the study period*. The use of pharmacological interventions for participants was assessed at baseline, 4 weeks, and 8 weeks.

### Sample size

Referring to a similar study related to using acupuncture on KOA [59], the effect size of VAS was 0.339, and the effect size of KOOS-subscales (pain, symptoms and quality-of-life) were 0.571, 0.322 and 0.517 [60]. To avoid underestimation of the sample size, the current study used the smallest effect size, that is, 0.322 with the power at 0.8, and the level of statistical significance at 0.05, the total number of participants estimated was 78. In consideration with a drop-out rate for around 15%, therefore, the number of participants for the current study was 90. The effect size was calculated using G-Power (version 3.1.9.4; Franz Faul, Universität Kiel, Düsseldorf).

### Statistical analysis

The descriptive analysis of the baseline characteristics was assessed by One-way analysis of covariance (ANOVA) for continuous data and by Kruskai-Wallis H Test for categorical data. The outcomes were analyzed using mixed model ANOVA (two-tailed) for time effect (Baseline, 4 weeks and 8 weeks after treatment) and group effect (G1, G2, and G3). Significance level was set at *p* = 0.05, 95% CI, and a Bonferroni correction was applied. Wilks’ Lambda was used for statistical significance analysis. The assumption of sphericity was tested by the Mauchly’s test, and the assumption of normality was tested using the Shapiro-Wilk test. Levene’s test was used for assessing equality of error variances. Post hoc analyses were conducted when a significant difference was found in the corresponding outcomes measures to identify the difference among the comparisons between groups. G1 and G2 participants’ responses of whether they had received real or placebo US-guided DN were assessed by Chi-square test. Statistical analyses were based on the Intention to treat (ITT) approach, including all randomized participants, a multiple imputation would be used if more than 5% drop-out [61, 62]. IBM SPSS (version 23; IBM Corp., Armonk, USA) was used for the data analysis by an independent co-investigator who was blinded to the treatment groups [63].

## Results

**Fig 1:**
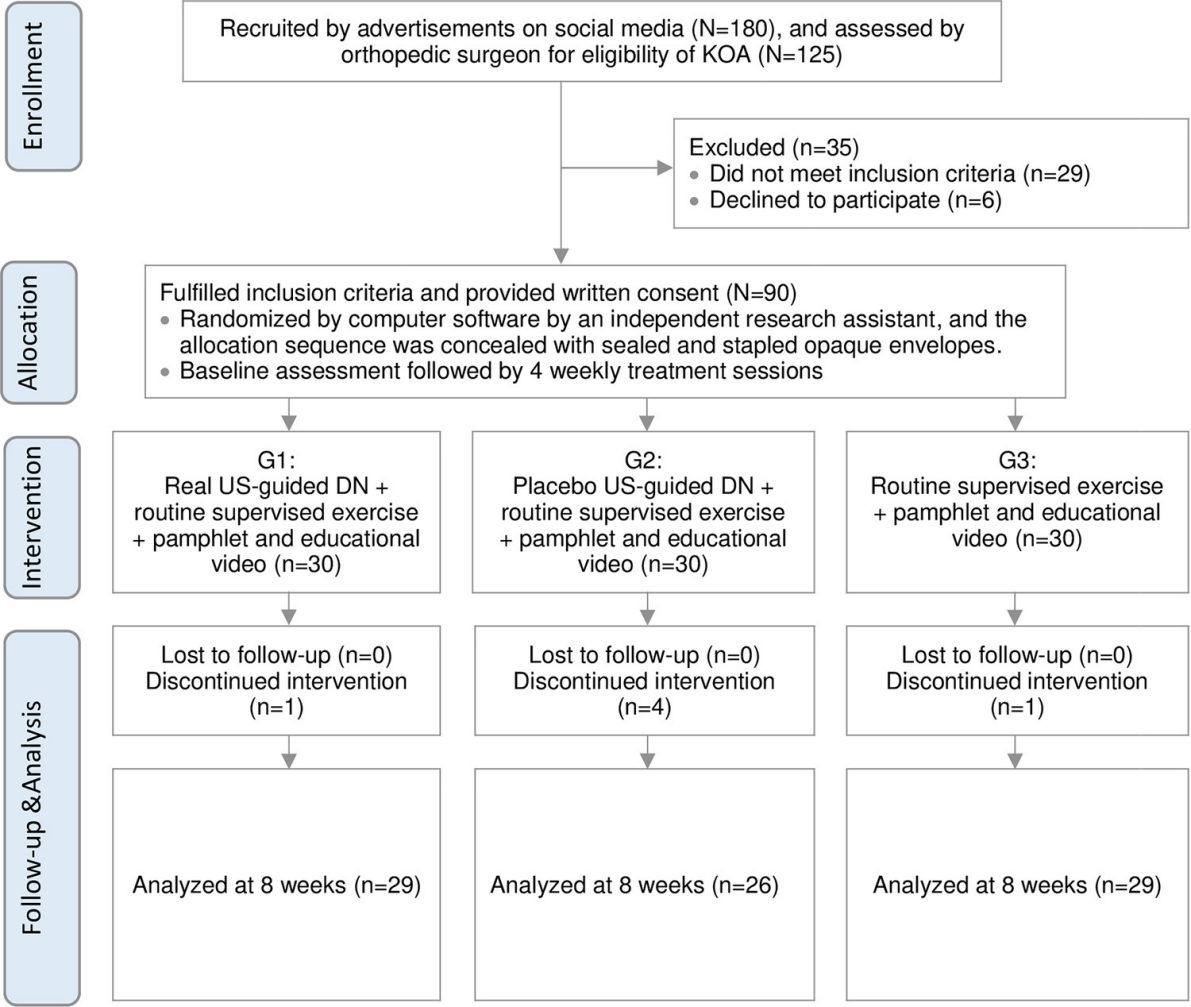
Consolidated Standards of Reporting Trials (CONSORT) flow diagram.

A total of one hundred and eighty participants were recruited at CIHE during July 2020 to January 2021. A total of ninety participants (25 male and 65 female) with a mean (SD) age of 61.26 (5.57) were eligible and included in this trial. Six participants (G1 = 1, G2 = 4, and G3 = 1) failed to complete the treatments due to fear of COVID-19 infection or a busy work schedule (**Fig 1**). The trial was stopped once the target number of participants was achieved by 16 March 2021. This was a complete case analysis due to the low number of dropouts with missing values totaled 3.843%. Therefore, the results were not further analyzed by ITT analysis. Moreover, we did not observe any clinically relevant differences between the three groups at the baseline, suggesting that no baseline imbalance was observed. The demographic data for the participants is summarized in **Table 1**.

**Table 1:** Characteristics of participants.

	G1	G2	G3
	N = 30	N = 30	N = 30
Age	60.56±5.93	61.23±5.49	61.97±5.39
Gender	Male = 9 (30.00%)	Male = 10 (33.30%)	Male = 6 (20.00%)
	Female = 21 (70.00%)	Female = 20 (66.70%)	Female = 24 (80.00%)
Height (m)	1.59±0.12	1.63±0.08	1.57±0.09
Weight (kg)	57.51±8.75	63.01±12.44	58.11±11.09
BMI (m/Kg^2^)	23.05±5.25	23.60±3.54	23.40±3.48
X-Ray findings	Grade I = 3	Grade I = 0	Grade I = 0
(KL Scale)	Grade II = 23	Grade II = 19	Grade II = 26
	Grade III = 4	Grade III = 11	Grade III = 4
History of Injury	0%	0%	0%
Duration of pain (months)	57.17±57.09	86.90±85.75	58.53±65.72
VAS	48.00±16.28	44.00±18.47	44.30±19.34
KOOS-pain	69.07±10.01	66.20±15.80	72.22±13.90
KOOS-symptoms	73.93±13.53	76.07±16.62	77.85±12.06
KOOS-QOL	48.75±19.37	58.13±21.66	55.00±15.86
Pharmacological intervention	2.76±10.66	1.04±2.75	2.34±11.19

Note: (Mean (SD) for continuous data and number of participants for categorial data)

After four weeks of intervention, outcomes revealed significant improvements in the VAS with an interaction between time and group effects (*p*<0.001) after a Bonferroni correction, therefore, a post-hoc test was done. US-guided DN showed significant improvement in 4 weeks (G1: -22.35, 95% CI [-31.80, -12.90], *p*<0.001) and in 8 weeks (G1: -33.35, 95% CI [-43.94, -22.75], *p*<0.001) (**Table 2A**). In addition, for between-group comparisons, G1 vs. G2 and G1 vs. G3 showed significant improvement in VAS measures of pain reduction at the 8-week follow-up (G1 vs. G2: -15.61, 95% CI [-25.49, -5.51], *p* = 0.001; G1 vs. G3: -19.90, 95% CI [-29.71, -10.08], *p*<0.001); while there was no significant difference in G2 vs G3 (-4.29, 95% CI [-14.38, 5.80], *p* = 0.905) (**Table 2B**). The improvements were likely clinically significant since they were over the minimum clinically important difference (MCID) of VAS, i.e., 19.9mm [59, 64].

**Table 2:** Comparisons of outcomes among G1, G2 and G3 after a Bonferroni correction.

a) Comparisons of change in outcomes over time for G1, G2 and G3							
Outcomes	Group	4-week vs Baseline Mean [95% CI]	P-value	8-week vs Baseline Mean [95% CI]	P-value	4-week vs 8-week Mean [95% CI]	P-value
VAS	G1	-22.35 [-31.80, -12.90]	<0.00*	-33.35 [-43.94, -22.75]	<0.00*	-11.00 [-31.80, -12.89]	<0.00*
	G2	-14.82 [-24.65, -4.96]	0.002*	-14.46 [-24.47, -4.46]	0.003*	-0.35 [-5.36, 6.05]	1.000
	G3	-12.76 [-22.34, -3.18]	0.006*	-10.66 [-20.48, -0.83]	0.030*	2.10 [-2.80, 7.00]	0.851
KOOS-Pain	G1	6.51 [1.02, 12.01]	0.016*	15.23 [0.63, 20.83]	<0.00*	8.72 [4.48, 12.96]	<0.00*
	G2	3.95 [-0.63, 8.53]	0.109	7.27 [2.50, 12.03]	0.002*	3.31 [-1.18, 7.80]	0.210
	G3	6.51 [1.02, 12.01]	0.016*	15.23 [9.63, 20.83]	<0.00*	8.72 [4.48, 12.96]	<0.00*
KOOS-Symptoms	G1	5.17 [0.32, 10.03]	0.034*	8.25 [2.74, 13.76]	0.002*	3.08 [-1.27, 7.43]	0.246
	G2	2.34 [-4.62, 9.29]	1.000	2.75 [-4.00, 9.50]	0.919	0.41 [-5.78 6.60]	1.000
	G3	3.82 [-0.89, 8.53]	0.169	-0.86 [-6.93, 5.20]	1.000	-3.82 [-8.53, 0.89]	0.145
KOOS-QOL	G1	2.16 [-2.27, 6.58]	0.712	4.28 [-0.05, 8.62]	0.054	2.13 [-1.57, 5.82]	0.490
	G2	0.48 [-6.52, 7.48]	1.000	0.96 [-7.97, 9.89]	1.000	0.48 [-6.29, 7.25]	1.000
	G3	-0.65 [-4.85, 3.56]	1.000	1.29 [-4.59, 7.17]	1.000	1.94 [-3.87, 7.75]	1.000
b) Between-group comparisons of change in outcomes for G1, G2 and G3 after a Bonferroni correction							
Outcomes	Time points	G1 vs G2 Mean [95% CI]	P-value	G1 vs G3 Mean [95% CI]	P-value	G2 vs G3 Mean [95% CI]	P-value
VAS	Baseline	4.00 [-7.39, 15.39]	1.000	3.70 [-7.69,15.09]	1.000	-0.30 [-11.69, 1.09]	1.000
	4-week	-4.26 [-15.13, 6.61]	1.000	-6.79 [-17.37, 3.78]	0.360	-2.53 [-13.41, 8.34]	1.000
	8-week	-15.61 [-25.49, -5.51]	0.001*	-19.90 [-29.71, -10.08]	<0.00*	-4.29 [-14.38, 5.80]	0.905
KOOS-Pain	Baseline	2.87 [-5.42, 11.16]	0.689	-3.15 [-11.44, 5.14]	0.638	-6.02 [-14.31, 2.27]	0.200
	4-week	4.35 [-3.40, 12.11]	0.377	1.25 [-6.29, 8.78]	0.918	-3.11[-10.86, 4.64]	0.606
	8-week	9.76 [2.38, 17.14]	0.006*	9.48 [2.31, 16.66]	0.006*	-0.28 [-7.65, 7.10]	0.996
KOOS-Symptoms	Baseline	-3.57 [-12.31, 5.17]	0.962	-2.90 [-11.41, 5.61]	1.000	0.67 [-7.84, 9.18]	1.000
	4-week	-1.51 [-7.62, 10.64]	1.000	-1.46 [-10.64, 7.62]	1.000	0.05 [-8.85, 8.94]	1.000
	8-week	1.65 [-7.61, 10.90]	1.000	5.93 [-14.94, 3.09]	0.336	4.28 [-4.74, 13.29]	0.748
KOOS-QOL	Baseline	9.38 [-2.39, 21.14]	0.145	6.25 [-5.52, 18.02]	0.418	-3.13 [-14.89, 8.64]	0.802
	4-week	3.20 [-6.67, 13.07]	0.720	-3.66 [-13.26, 5.94]	0.635	-6.86 [-16.74, 3.01]	0.227
	8-week	0.17 [-10.29, 10.32]	1.000	-5.39 [-15.41, 4.64]	0.409	= 5.40 [-15.71, 4.90]	0.427

Note: * represented P-value significant for <0.05 with Bonferroni correction. Abbreviations: CI, confidence interval; G1, group 1; G2, group 2; G3, group 3; KOOS, Knee Injury and Osteoarthritis Outcome Score; QoL, quality-of-life; SD, standard deviation; VAS, Visual Analogue Scale.

Moreover, G1 also showed significant improvement in KOOS-pain at 4 weeks after a Bonferroni correction (G1: 6.51, 95% CI [1.02, 12.01], *p* = 0.016) and 8 weeks (G1: 15.23, 95% CI [9.63, 20.83], p<0.001) (**Table 2A**). Similarly, KOOS-symptoms and KOOS-QoL showed significant improvement at 4-week and 8-week follow-up. In the between-group analysis, G1 achieved significant improvement in KOOS-pain at the 8-week follow-up compared to G2 and G3 (G1 vs. G2: MD = 9.76, 95% CI [2.38, 17.14], *p* = 0.006; G1 vs. G3: MD = 9.48, 95% CI [2.31, 16.66], *p* = 0.006) (**Table 2B**). The improvements were likely clinically significant since they were over the MCID of KOOS-pain, i.e., scores of 9.3 [64]. However, there was no significant difference for KOOS-pain between G2 and G3 (MD = -0.28, 95% CI [-7.65, 7.10], *p* = 0.996). In addition, all three groups showed no significant differences in group effects for the KOOS subscale-symptoms and KOOS-QoL (**Table 2B**).

The distributions of sonographic findings and tender spots upon palpation in physical examination for the participants are shown in the **S1 Table**. The selection of puncture sites was based on the findings with both abnormal sonographic findings and tenderness upon palpation. The number of tender spots was 2.73 (1.01) in G1 and 2.60 (0.72) in G2. The needles were used for total 4 sessions in G1 = 12.97 (4.49) and G2 = 12.31 (3.38), i.e., in average 3 needles per session in **S2 Table**.

The most common findings in the sonography examination among the participants were mucoid degeneration (G1 = 24, G2 = 26), then hypo-echogenicity (G1 = 2, G2 = 3), lastly hyper-echogenicity (G1 = 4 and G2 = 1) in **S3 Table**.

The means and standard deviations of exercise compliance are provided in **S4 Table**. The interactions between time and group effects were not statistically significant (*p* = 0.077). In addition, the means and standard deviations of pharmacological intervention in the three groups at different times are shown in **S5 Table**. The interactions between the time and group effects were also not statistically significant (*p* = 0.633). The G2 participants had no significant difference of the perceptions as those of G1 with p = 0.128.

### Adverse events

There were no occurrences of infection or adverse events during the trial.

## Discussion

### Main findings

The findings of this double-blind RCT are the first to validate the benefits of US guidance in combination with DN therapy for patients with KOA. US-guided DN therapy demonstrated a statistically significant reduction in knee pain and dysfunction relative to the baseline in 4-week and 8-week follow-ups. Within the three types of interventions applied, we found statistically significant difference between G1 and G2 for pain reduction at 8 weeks (15.61mm on VAS) and reduction of 19.90mm between G1 and G3. In addition, we found 9 point-difference for KOOS-pain subscale at 8 weeks. However, there was no statistically significant differences for KOOS symptoms or quality-of-life.

To minimize sampling bias, a double-blind randomized controlled trial was employed. In addition, participants were recruited from the general public openly by paper and digital advertisements in social media, which may have minimized selection bias, thereby improving the generalizability of the study findings. On the other hand, recruiting via advert to open public might have introduced different selection bias as participants often very different to standard clinical cohorts. The inclusion and exclusion criteria can provide illustrate the characteristics of the participants.

### Miss data and ITT analysis

Missing data may compromise the inferences from RCTs if such data are handled inappropriately [61]. ITT is a valid method for addressing missing data and commonly used in RCT [65]. In the current study, missing values totaled 3.843%. As the missing data did not exceed 5%, literature recommends that ITT analysis is not applicable [61].

### Comparing findings with other studies

Traditionally, the selection of a puncture site has been determined by the patients’ symptoms or the presence of myofascial trigger points (MTrPs), hard, well-defined palpable nodules discovered in the process of physical examination [66–68]. Once desired twitch response has been obtained, the needle is moved in a piston-like motion [66–68]. Without US guidance; however, the operating physiotherapist is unable to visualize the anatomical structure that is being targeted. Consequently, needle-puncturing and maneuvering may be misguided by an LTR that does not originate from within the problematic tissue. Furthermore, some patients may respond with an LTR so early that the advancement of the needle must be stopped, though the target is located within a lower layer. Sources of pain may stem from deeper structures, such as the hyaline cartilage, joint capsule, articular ligament, or articular retinaculum [66–68].

**Fig 2:**
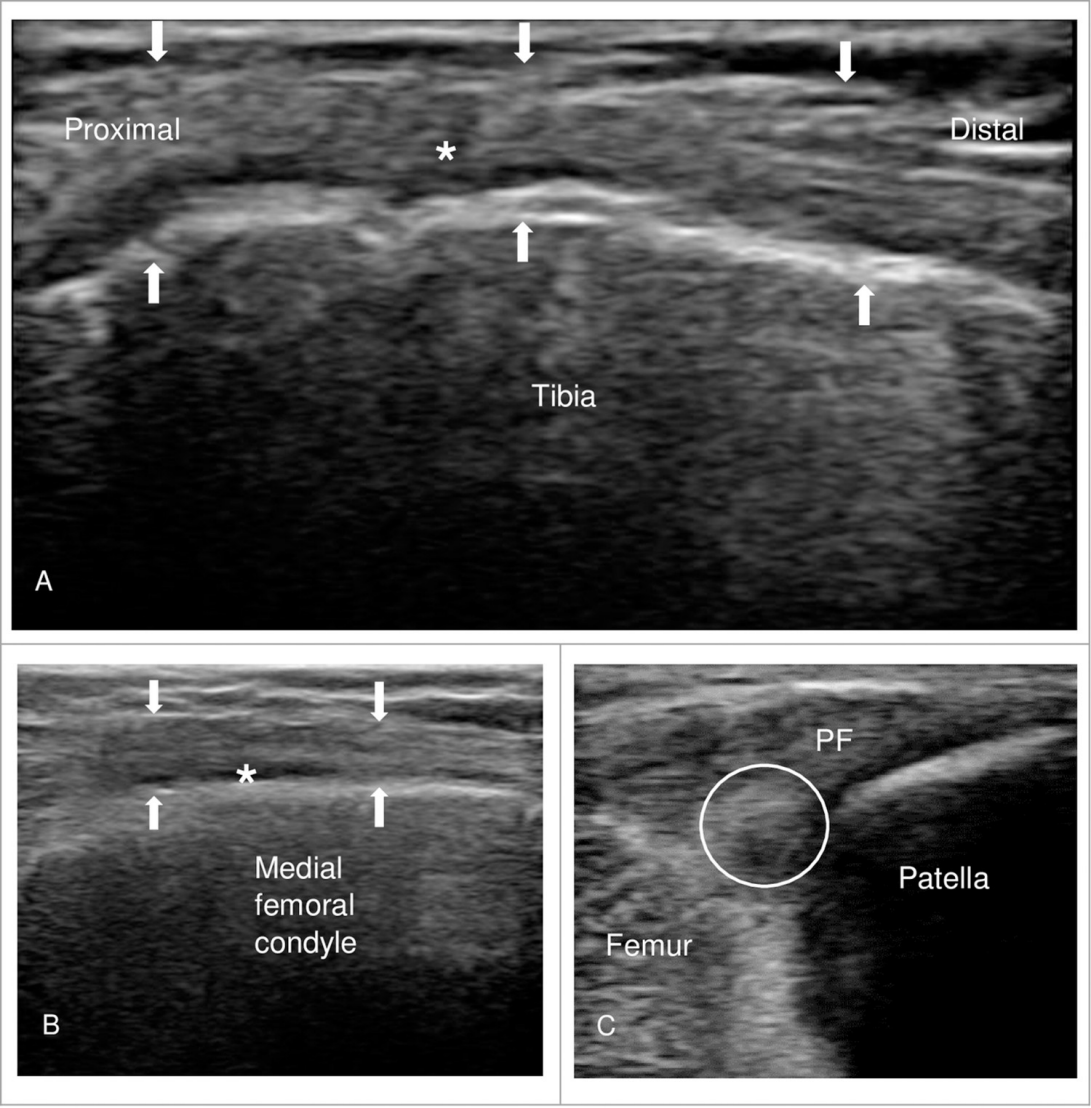
Sonographic examination images of a patient with KOA. (A) Coronal plane of the medial collateral ligament (MCL: white arrow) displaying mucoid degeneration (white star*); (B) Hypoechoic appearance of the medial collateral ligament (white arrow) in transverse plan displaying effusion (white star*); (C) Longitudinal view of the patellofemoral ligament (PFL) displaying hyperechoic change in the region of patella (white circle).

**Fig 3:**
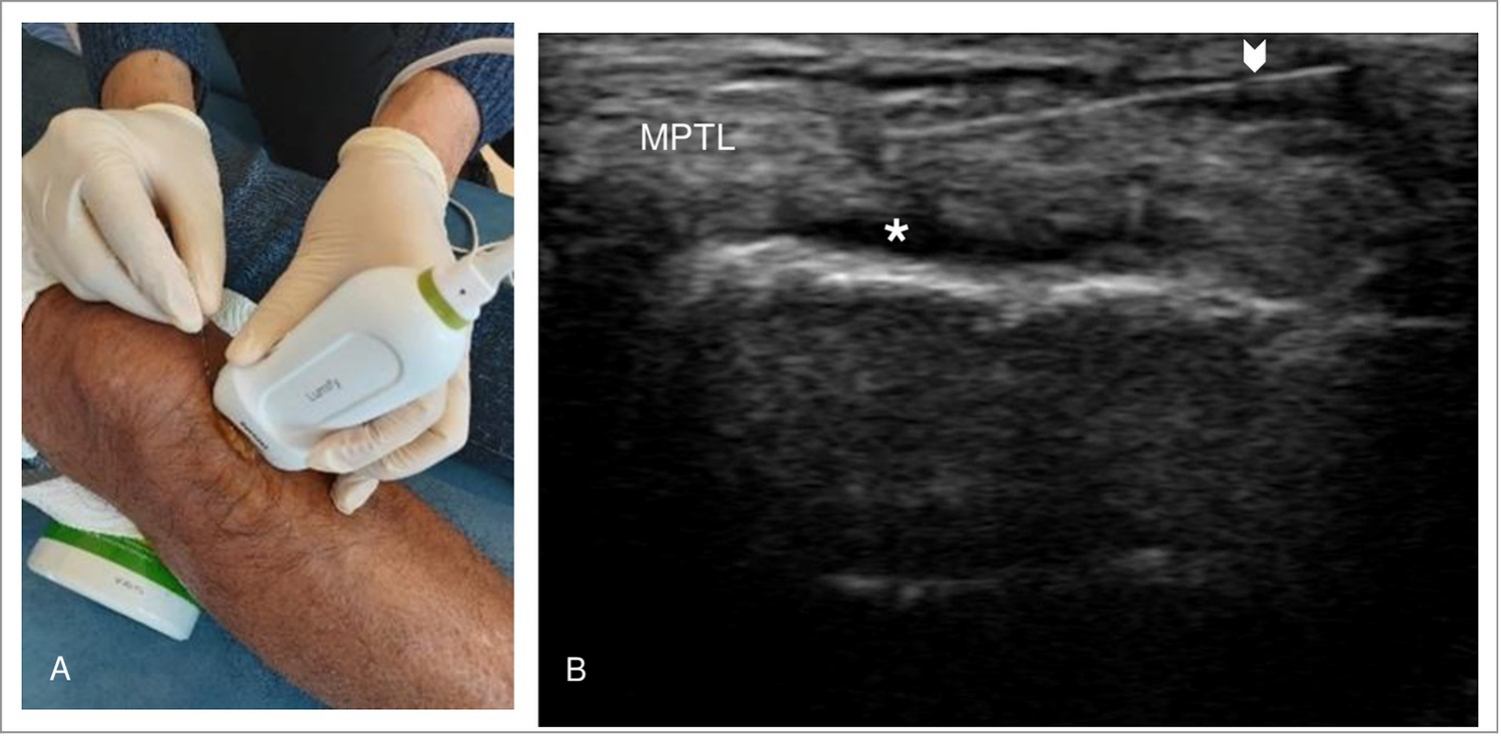
Application of US-guided DN in patients with mucoid degeneration of medial patellotibial ligament (MPTL). (A) Positioning of the DN and transducer in the application of US-guided DN; (B) Longitudinal view of the medial patellotibial ligament (MPTL) with hyperechoic change (white star*). The needle with reverberation artifact was displayed by arrowhead.

DN with US provides an initial awareness of an anatomical abnormality within the knee structure. Heterogeneous echogenicity may indicate hypoechoic swelling, mucoid degenerative change [18] and/or a superimposed interstitial tear [18]. Hyperechoic foci in sonography may signify the presence of calcium pyrophosphate dihydrate crystals [18]. In this RCT, sonographic examination of patients in G1 revealed areas of hyperechogenicity (13.3%), hypoechogenicity (6.6%), and mucoid degeneration (80%) (**Fig 2**) The puncture site was then selected if the abnormal finding in the sonographic image correlated with the patients’ symptoms and physical examination. Subsequently, sonography permitted visualization of the needle’s advancement and its action of being passed in-and-out of the targeted tissue (**Fig 3**).

As noted, US-guided DN has shown improvement in tendinopathy of the shoulder [44, 45, 69] while regaining the echogenicity of the previously hypoechoic tissue in the supraspinatus tendon [41]. The effect of US-guided DN on KOA in this study is postulated to have a similar pattern of physiological response and recovery within the treated soft tissues. The anatomical visualization enabled using US-guidance permits a more accurate targeting than that possible by DN alone and suggests that it more effectively promotes healing, reduces pain, and improves functional ability in patients with KOA.

### Implications

The findings from this study may provide scientific evidence for physiotherapist to apply US-guided DN to treat patients with KOA. In addition, this study is the first double-blinded RCT to provide scientific data to support the effectiveness of US-guided DN with exercises is more favorable than DN with exercise or exercise alone in patients with KOA. This study may enlighten further studies in the development of pain management in other painful musculoskeletal conditions.

### Limitation

In this study, only the short-term effects of US-guided DN in KOA were investigated; further research is necessary to identify the longer-term effects. The effects investigated were a combined effect of US-guided DN and exercises and the G3 received exercise therapy rather than no treatment. Therefore, the study may be underpowered to show the sole effect of US-guided DN. Additionally, in view of the nature of intervention, the blinding to operative physiotherapist is nearly impossible that may cause performance bias. Finally, the recruited participants presented with mild to moderate degenerative changes in their X-ray reports using the KL scale of Grade I to Grade III. Consequently, the results of this study may not be applicable to cases of severe degenerative changes having a Grade IV in the KL scale.

## Conclusion

The effectiveness of DN can be improved through US-guided needle advancement that more accurately targets anatomical tissue than traditional DN. The findings from the current study provide evidence to support the adaptation of US-guided DN in treating patients with KOA. This new technique may assist the clinical practice of physiotherapists. Overall, it may help to reduce pain experienced by patients with KOA.

## Supporting information

S1 Checklist (DOCX)

S1 File Knee sonographic examination procedure being adopted with reference to Jacobson (2018). (PDF)

S2 File (PDF)

S3 File (DOC)

S1 Table The raw data for the sonographic examination and painful spots during palpation in physical examination for the selection of sites for US-guided DN. (PDF)

S2 Table Number of tender spots and number of needles used. (PDF)

S3 Table Sonographic findings and distribution of tender spots for US-guided DN (G1) and placebo US-guided DN (G2). (PDF)

S4 Table Exercise compliance for the three groups at different time points. (PDF)

S5 Table The mean of pharmacological intervention for the three groups at different time points. (PDF)
