# Supplementary material for: Ultrasound-guided dry needling versus traditional dry needling for patients with knee osteoarthritis: A double-blind randomized controlled trial
Source: PLoS One. 2022 Sep 30;17(9):e0274990. doi: 10.1371/journal.pone.0274990 (PMC9524650; doi:10.1371/journal.pone.0274990)
Supplement: S1 Checklist — (DOCX) [file pone.0274990.s001.docx]

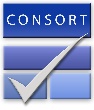
CONSORT 2010 checklist of information to include when reporting a randomised trial*

| Section/Topic | Item No | Checklist item | Reported on page No |
| --- | --- | --- | --- |
| Title and abstract | | | |
|  | 1a | Identification as a randomized trial in the title | Page 3, line 1-2 |
|  | 1b | Structured summary of trial design, methods, results, and conclusions (for specific guidance see CONSORT for abstracts) | Page 3, lines 1-23 |
| Introduction | | | |
| Background and objectives | 2a | Scientific background and explanation of rationale | Pages 4-5, lines 28-71 |
|  | 2b | Specific objectives or hypotheses | Page 5, lines 73-74 |
| Methods | | | |
| Trial design | 3a | Description of trial design (such as parallel, factorial) including allocation ratio | Page 5, lines 78-80 |
|  | 3b | Important changes to methods after trial commencement (such as eligibility criteria), with reasons | N/A |
| Participants | 4a | Eligibility criteria for participants | Page 6, lines 95--114 |
|  | 4b | Settings and locations where the data were collected | Page 5, line 92-93 |
| Interventions | 5 | The interventions for each group with sufficient details to allow replication, including how and when they were actually administered | Pages 7-8, lines 138-194 |
| Outcomes | 6a | Completely defined pre-specified primary and secondary outcome measures, including how and when they were assessed | Page 8, 196-213 |
|  | 6b | Any changes to trial outcomes after the trial commenced, with reasons | N/A |
| Sample size | 7a | How sample size was determined | Page 9, lines 216-220 |
|  | 7b | When applicable, explanation of any interim analyses and stopping guidelines | Page 5, lines 92-93 |
| Randomization: |  |  |  |
| Sequence generation | 8a | Method used to generate the random allocation sequence | Pages 5-6, lines 115-136 |
|  | 8b | Type of randomization; details of any restriction (such as blocking and block size) | N/A |
| Allocation concealment mechanism | 9 | Mechanism used to implement the random allocation sequence (such as sequentially numbered containers), describing any steps taken to conceal the sequence until interventions were assigned | Page 6, lines 121-123 |
| Implementation | 10 | Who generated the random allocation sequence, who enrolled participants, and who assigned participants to interventions | Page 6, lines 119-121 |
| Blinding | 11a | If done, who was blinded after assignment to interventions (for example, participants, care providers, those assessing outcomes) and how | Page 7, lines 134-136 |
|  | 11b | If relevant, description of the similarity of interventions | N/A |
| Statistical methods | 12a | Statistical methods used to compare groups for primary and secondary outcomes | Pages 9-10, lines 224-233 |
|  | 12b | Methods for additional analyses, such as subgroup analyses and adjusted analyses | N/A |
| Results | | | |
| Participant flow (a diagram is strongly recommended) | 13a | For each group, the numbers of participants who were randomly assigned, received intended treatment, and were analyzed for the primary outcome | Page 10, Lines 236-238 |
|  | 13b | For each group, losses and exclusions after randomization, together with reasons | Page 10, lines 237-239 |
| Recruitment | 14a | Dates defining the periods of recruitment and follow-up | Page 10, lines 236-240 |
|  | 14b | Why the trial ended or was stopped | Page 10, lines 239-240 |
| Baseline data | 15 | A table showing baseline demographic and clinical characteristics for each group | Page 10, Line 244 (**TABLE 1)** |
| Numbers analyzed | 16 | For each group, number of participants (denominator) included in each analysis and whether the analysis was by original assigned groups | Page 10, **FIGURE 3** |
| Outcomes and estimation | 17a | For each primary and secondary outcome, results for each group, and the estimated effect size and its precision (such as 95% confidence interval) | Pages 12-14 (**TABLES 2a and 2b, eTables 1-5** ) |
|  | 17b | For binary outcomes, presentation of both absolute and relative effect sizes is recommended | N/A |
| Ancillary analyses | 18 | Results of any other analyses performed, including subgroup analyses and adjusted analyses, distinguishing pre-specified from exploratory | N/A |
| Harms | 19 | All important harms or unintended effects in each group (for specific guidance see CONSORT for harms) | Pages 16-17, lines 362-371 |
| Discussion | | | |
| Limitations | 20 | Trial limitations, addressing sources of potential bias, imprecision, and, if relevant, multiplicity of analyses | Page 15, lines 311-316 |
| Generalizability | 21 | Generalizability (external validity, applicability) of the trial findings | Page 15, lines 311-316 |
| Interpretation | 22 | Interpretation consistent with results, balancing benefits and harms, and considering other relevant evidence | Page 15-16, lines 301-354 |
| Other information | | |  |
| Registration | 23 | Registration number and name of trial registry | Page 5, lines 82-83 |
| Protocol | 24 | Where the full trial protocol can be accessed, if available | NA |
| Funding | 25 | Sources of funding and other support (such as supply of drugs), role of funders | Page 18, lines 418-422 |

*We strongly recommend reading this statement in conjunction with the CONSORT 2010 Explanation and Elaboration for important clarifications on all the items. If relevant, we also recommend reading CONSORT extensions for cluster randomised trials, non-inferiority and equivalence trials, non-pharmacological treatments, herbal interventions, and pragmatic trials. Additional extensions are forthcoming: for those and for up to date references relevant to this checklist, see [www.consort-statement.org](http://www.consort-statement.org).
