# Supplementary material for: Ultrasound-guided dry needling versus traditional dry needling for patients with knee osteoarthritis: A double-blind randomized controlled trial
Source: PLoS One. 2022 Sep 30;17(9):e0274990. doi: 10.1371/journal.pone.0274990 (PMC9524650; doi:10.1371/journal.pone.0274990)

S1 File. Knee sonographic examination procedure being adopted with reference to Jacobson (2018).

1. Anterior and medial patellofemoral compartment

Anterior patella-femoral compartment	Quadriceps muscles and quadriceps tendon
	Patella
	Patella tendon
	Patella retinaculum
	Suprapatellar plica
	Suprapatellar recess
	Medial and lateral recess
	Medial patellofemoral ligament
	Medial patellotibial ligament
	Joint effusion over quadriceps fat pad, pre-patella fat pad, superficial infrapatellar bursa, deep infrapatellar bursa, Hoffa's fatpad
	Femoral articular cartilage included subchondral bone architectural changes and cartilage loss
Medial	Medial collateral ligament
	Medial meniscus
	Pes anserinus: Sartorius, gracillis, semitendinousus and the underneath bursa

2. The most common anatomical locations of pain in OAK patients

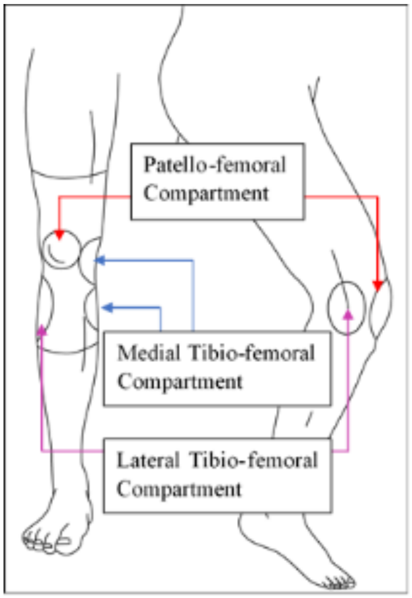

Figure 1. The most common anatomical locations of pain in OAK patients (Sengupta et al. 2006).

Figure 2A. shows the position of anterior knee US scanning for the Sagittal view and 2B shows the quadriceps tendon (black arrowheads), quadriceps fat pad (Q), prefemoral fat pad (PF), and collapsed joint recess (white arrow), F=femur and P=patella (Jacobson 2018).

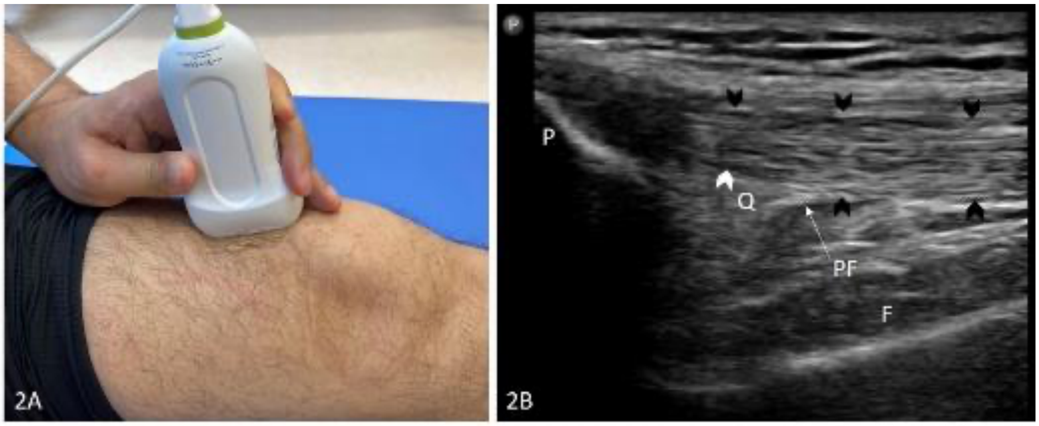

Figure 3A and 3B. Short axis of quadriceps femoris (Figure 3A) and the quadriceps tendon (arrowheads) (Figure 3A).

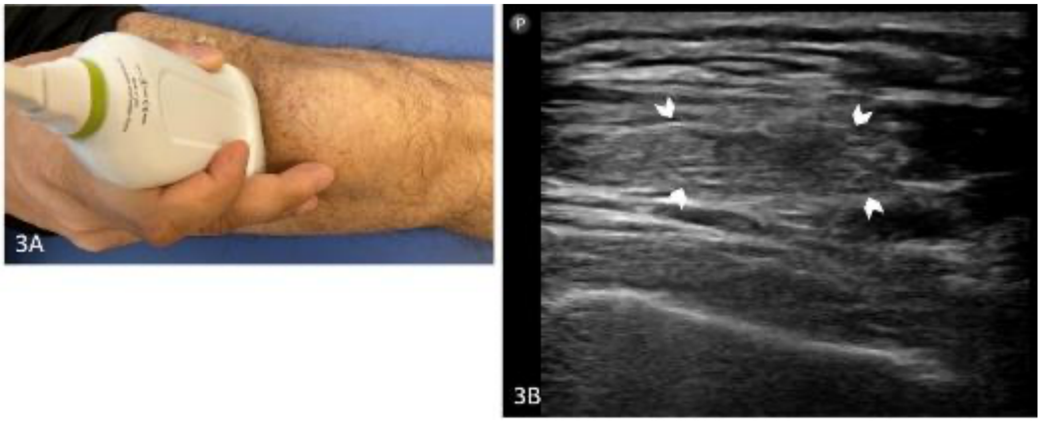

Figure 4A and 4B. US scanning to patella tendon in sagittal view (Figure 4A) and the patella tendon (arrowheads) and Hoffa fat pad (H). P=patella and T=tibia in (Figure 4B).

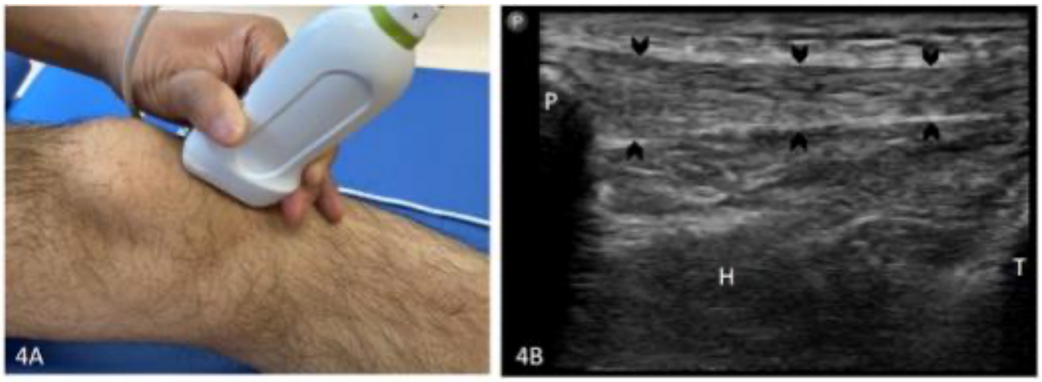

Figure 5A. US scanning to patella tendon in transverse view (Figure 5A) and the patella tendon (arrowheads) and Hoffa fat pad (H) in Figure 5B.

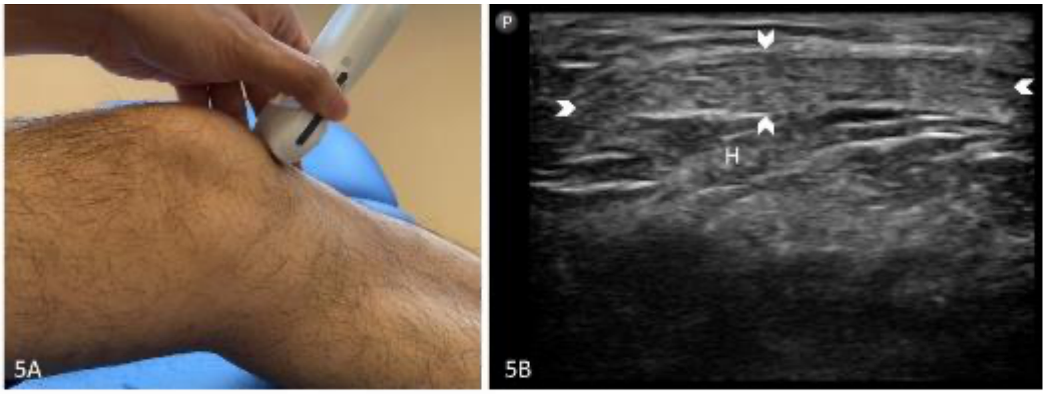

Figure 6A. US scanning to patellofemoral tendon in longitudinal view (Figure 6A). The medial knee joint recess (white arrow) and the arrowheads show the patellofemoral ligament (Figure 6B) and position of patellofemoral ligament in medial knee (Figure 6C). F=femoral condyle, P=patella.

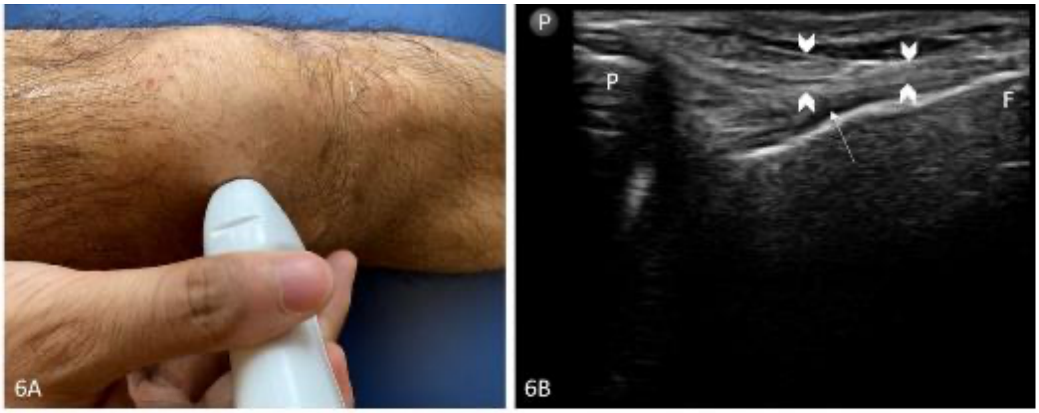

Figure 6C. The location of the medial patellotibial ligament (MPTL), medial patellofemoral ligament (MPFL) and it runs obliquely and transverse origin from the femoral epicondyle and adductor tubercle. Around the vastus medialis oblique muscle (VMO), the medial collateral ligament (MCL), and the adductor magnus tendon (AMT) are outlined with dotted lines (Jacobson 2018).

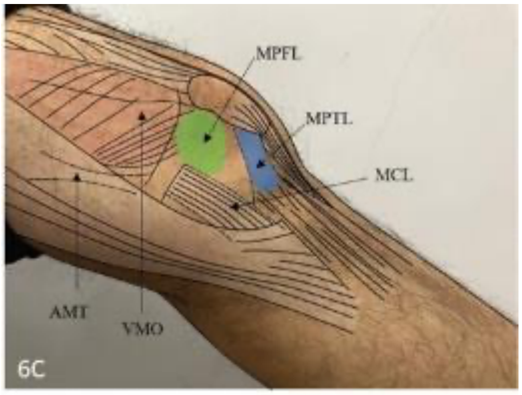

Figure 7A. US scanning to patellotibial tendon in longitudinal view (Figure 7A) and position of patellotibial ligament in medial knee (Figure 6C). The arrowheads show the patellotibial ligament (Figure 7B). T=tibial condyle, P=patella.

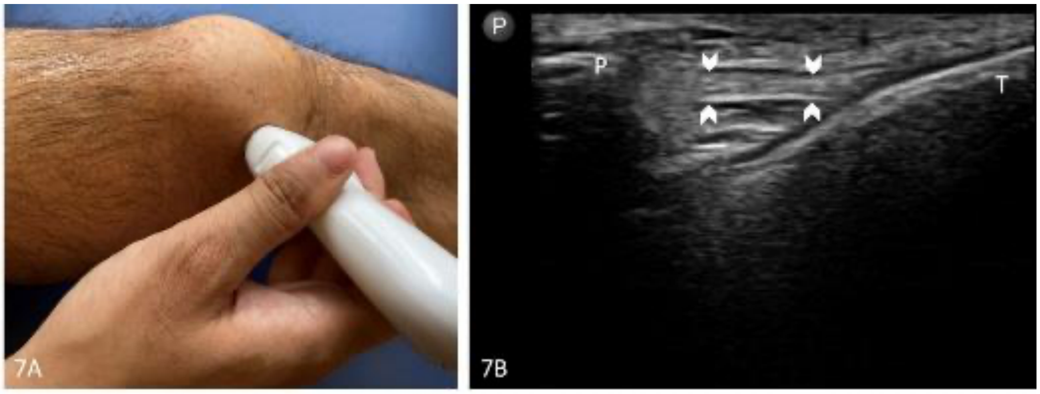

Figure 8A. Trochlear and femoral condyle cartilage. Figure 8A shows hypoechoic hyaline cartilage (arrowheads). LC=lateral femoral condyle, MC=medial femoral condyle, P=patella.

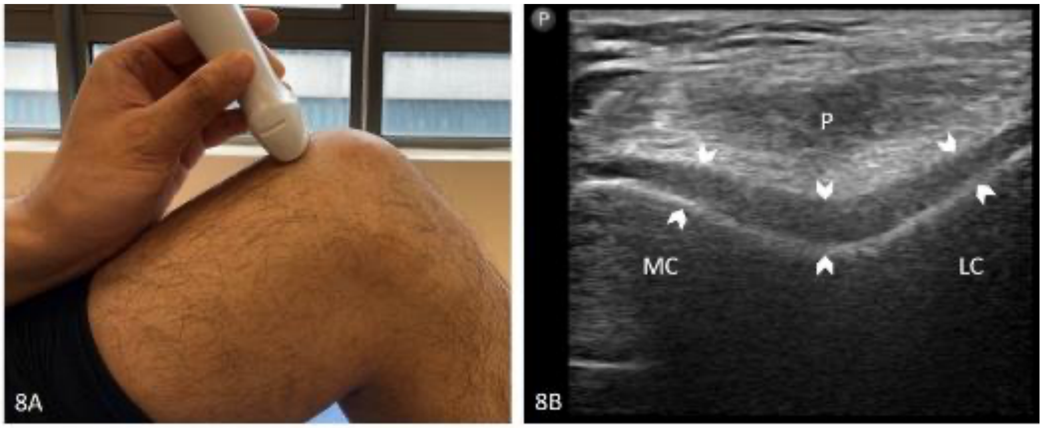

Figure 9A. Medial knee sonography evaluation in coronal plane (Figure 9A). Coronal imaging at the medial joint line is shown in (Figure 9B) the superficial (arrows) and deep (arrowheads) layers of the medial collateral ligament, and medial meniscus (white arrow). F=femur, T=tibia.

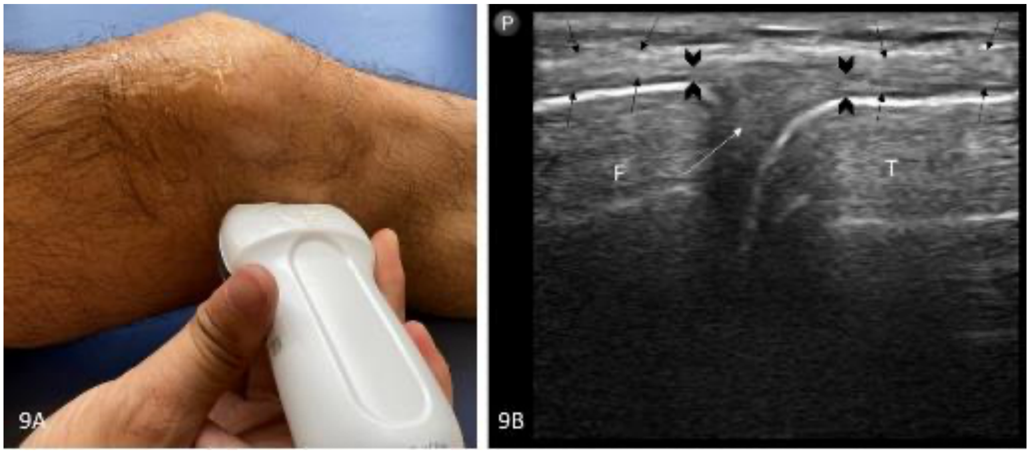

Figure 10A. Sonography examination of the medial collateral ligament in transverse plane (Figure 10A). Transverse imaging shows the medial collateral ligament (arrowheads). F=femur.

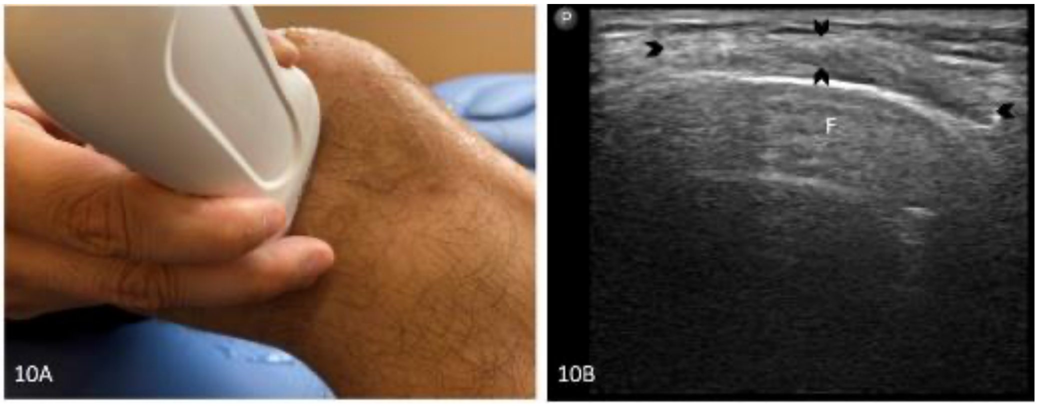

Figure 11A. Distal medial collateral ligament and pes anserinus. Coronal imaging distal to knee joint shows (Figure 11A), the superficial layer of the medial collateral ligament (arrowheads) and the tendons of the pes anserinus (arrows) (T=tibial metaphysis). Sonographic imaging in long axis shows the semitendinosus proximal to pes anserinus (arrowheads).

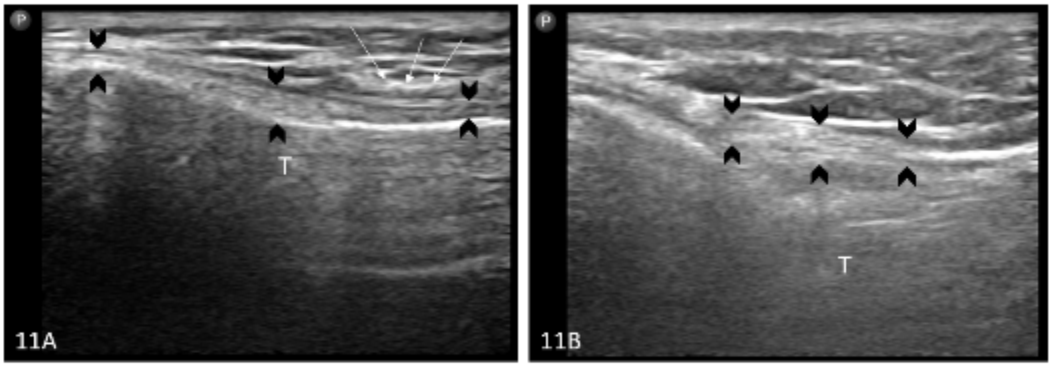

Supplement: S1 File — (PDF) [file pone.0274990.s002.pdf]
