# Supplementary material for: Ultrasound-guided dry needling versus traditional dry needling for patients with knee osteoarthritis: A double-blind randomized controlled trial
Source: PLoS One. 2022 Sep 30;17(9):e0274990. doi: 10.1371/journal.pone.0274990 (PMC9524650; doi:10.1371/journal.pone.0274990)
Supplement: S2 File — (PDF) [file pone.0274990.s003.pdf]

Request for Permission to Publish Content under CC-BY License

Dear Rights Holder or Representative,

I have submitted a paper for publication in a PLOS journal, and wish to include the content listed below in the paper. I'm hereby requesting your (or your company's or institution's) permission to include the content in my paper. Please note that all PLOS journals are published under a Creative Commons Attribution License (CC BY), which allows for unrestricted use and distribution, even commercial, as long as attribution is given to the creator or rights holder of the content. See https://creativecommons.org/licenses/by/4.0/.

To grant me permission to use the content in my PLOS paper, please fill in the information below and then scan the completed form and send it to me at my email address.

Thank you.

My name:

Johnson C.Y. Pang

My email address:

jpang@cihe.edu.hk

Description of the content which I'm seeking permission to use (citation and/or title, and pasted screen shot, if applicable):

Figure 2A, 2B, 3A, 3B, 4A, 4B, 5A, 5B, 6A, 6C, 7A, 7B, 8A, 8B, 9A, 9B, 10A, 10B, 11A, 11B in Appendix VI.

Link to the Content:

https://www.protocols.io/view/title-ultrasound-guided-dry-needling-versus-tradit-cgh5tt

* * *

On behalf of myself or the rights holder, I hereby grant the permission sought herein.

Signature of Party Granting Permission:

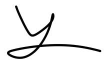

Date:

13/09/2022

Printed Name and Title:

Johnson C.Y. Pang
