# Supplementary material for: Ultrasound-guided dry needling versus traditional dry needling for patients with knee osteoarthritis: A double-blind randomized controlled trial
Source: PLoS One. 2022 Sep 30;17(9):e0274990. doi: 10.1371/journal.pone.0274990 (PMC9524650; doi:10.1371/journal.pone.0274990)
Supplement: S3 File — (DOC) [file pone.0274990.s004.doc]

**Study Protocol**

**Study title:** Effectiveness of ultrasound-guided dry needling on pain and functional disability for knee osteoarthritis: a double-blind, randomized controlled trial

**Study Number:** HSEARS20200314001

**Applicant information:**

Dr. Johnson C.Y. Pang, School of Health Sciences, Caritas Institute of Higher Education; [jpang@cihe.edu.hk](mailto:jpang@cihe.edu.hk)

**Other investigators and affiliations (list all):**

Professor Amy S.N. Fu, PT, PhD, Department of Rehabilitation Sciences, The Hong Kong Polytechnic University; [amy.fu@polyu.edu.hk](mailto:amy.fu@polyu.edu.hk)

Dr. Stanley K.H. Lam, MBBS, MScSEM, The Hong Kong Institute of Musculoskeletal Medicine; Department of Family Medicine, The Chinese University of Hong Kong; Department of Family Medicine, The University of Hong Kong; [drlamkh@gmail.com](mailto:drlamkh@gmail.com)

Dr. B. Peng, MD, MS, Department of Rehabilitation Medicine, Sichuan Academy of Medicine Sciences; [pblucky63@gmail.com](mailto:pblucky63@gmail.com)

Dr. Allan C.L. Fu, PT, PhD, Discipline of Physiotherapy, Sydney School of Health Sciences, The University of Sydney; Musculoskeletal Research Hub, Charles Perkins Centre, The University of Sydney; Email: [allan.fu@sydney.edu.au](mailto:allan.fu@sydney.edu.au)

**Purpose of the project:**

The aims of this study are to investigate the effects of dry needling (DN) with ultrasound (US)-guided and with placebo US-guided real-time US imaging on patients with knee osteoarthritis (KOA). Control group will be used to compare the effects of routine exercise therapy. The findings obtained from this study will help to improve the efficient in applying DN technique to KOA patients.

**The type of research project:**

This is a double-blind randomized controlled trial.

**The location where the project is going to be conducted:**

All procedures will be conducted at the laboratory of Caritas Institute of Higher Education.

**Funding agency and grant number**:

This study is funded by the grant from the Caritas Institute of Higher Education for publication with reference code: IDG200101.

**Background:**

Nowadays, aging population is the world-wide facing problem (WHO, 2018) and KOA is the most common type of osteoarthritis (OA) in aging population (Andrianakos et al., 2003). Its prevalence is rising along with the increasing aging population (Felson et al., 2000). Pain and dysfunction are the two main clinical symptoms. Pain (Naredo et al., 2005; Rathwa, Prajapati, & Deepak, 2019; Roos & Toksvig-Larsen, 2003) impaired muscular stabilization (Brandt et al., 2000; Cowan, Bennell, Hodges, Crossley, & McConnell, 2001), reduced range of motion (Steultjens, Dekker, van Baar, Oostendorp, & Bijlsma, 2000), and functional disability (Granvir, 2015; Harkey et al., 2019; Rathwa et al., 2019; Sadatsuki et al., 2019).

More recently, DN has been used in treating musculoskeletal pain with success. The technique of DN involves needle insertion into the target (in most condition, the most painful site) sites, then repeated puncture aiming to stimulate an inflammatory healing response. The needling may disrupt the collagen fibres at the target structure that may cause local haemorrhage and such micro-inflammation may lead to granulation tissue formation and enhanced tendon strength (Davidson & Jayaraman, 2011).

US imaging has been used as a guided intervention including DN treatment that passing the needle repeatedly through the abnormal tendon substance for a period of time (Daftary & Karnik, 2015; James et al., 2007, Lungu et al., 2018). The selection of needling site is based on anatomical lesion, and manual manipulation will be applied to the needles repeatedly in DN. The advantage of US imaging obviously allows more histological and radiological of the processes involved in soft-tissue injury that leads to novel targeted treatment (Davidson & Jayaraman, 2011). US-guided DN has been investigated and showed improvement in the efficacy of management such as rotator cuff tendinopathy (Fabbro et al., 2012), chronic Achilles tendinopathy (Yeo, Kendall & Jayaraman, 2016) and patellar tendinopathy (Colberg & Henderson, 2017) which allows more precise location to the target anatomical structures; and let the therapists to understand more about the current condition of the target structures (Lindseth et al., 2013; Mcnally, 2011).

To the best of my knowledge, there is no study in the literature that researches the impact of DN with and without US-guidance on pain management and functional recovery in patients presenting with KOA. The current study will include a control group to contrast the condition of natural healing with routine exercise and knee care against the interventions of DN with and without US guidance. An effective physiotherapy for patients with KOA has been recommended at least 4 sessions of weekly treatment (Vaishya et al. 2016).

**Null hypotheses:**

There are two null hypotheses to be tested in this study:

Null hypothesis 1: There would be no significant differences on pain modulation among the subject groups receiving US-guided DN (G1), placebo US-guided DN group (G2) and the control (G3).

Null hypothesis 2: There would be no significant difference on modulation on functional scores among the subject groups receiving US-guided DN (G1) compared to those in the placebo US-guided DN group (G2) or the control group (G3).

**Outcomes measures:**

Primary outcomes:

1. Pain level of the KOA patients will be assessed by Visual-Analogue Scale (VAS) (Alghadir et al., 2018; Boonstra et al., 2008)
2. The functional mobility of the KOA patients will be assessed by the Knee Injury and Osteoarthritis Outcome Score (KOOS)-short form (Roos et al., 2003; Cheung, Ngai and Ho., 2016)

Secondary outcomes:

1. Compliance of Exercise

Exercise compliance for participants was assessed at 4 weeks and 8 weeks by an independent assessor. The duration of exercise was assessed by hour per week. (Appendix VII).

1. Medication Utilization During the Study Period

The use of pharmacological interventions for participants was assessed at baseline, 4 weeks, and 8 weeks.

**Methodology:**

1. Study Design

A randomized, double-blinded, controlled, parallel group, repeated-measures design will be used. Ethical approval will be obtained from The Hong Kong Polytechnic University and Caritas Institute of Higher Education. The trial will be conducted in accordance with the Declaration of Helsinki.

1. Study subjects

Based on the effect sizes from previous relevant studies, the sample size has been calculated and 90 patients with KOA will be recruited from the general public with the physiotherapy referral from a doctor with the diagnosis of KOA and screened by knee X-ray with the Kellgren and Lawrence (KL) scale (Petersoon et al., 1997). Their age, gender, body mass index (BMI), unilateral or bilateral knees involved, duration of knee pain and any history of knee injury will be recorded as baseline information. The current study targets in mild to moderate degenerative KOA with XR showing with the KL scale (Petersoon et al., 1997) grade I and grade III (which is most beneficial to conservative treatment) (Appendix III). KL scale was proposed in 1957 and accepted by the WHO in 1961, it has been widely used in grading the severity of KOA (Samuel & Kanimozhi, 2019). All the classification of XR with the KL scale in this study will be done by a physiotherapist who has more than 10 years experience in musculoskeletal conditions and training in interpretation of knee XR with the classification of KL scale. All the subjects meet the inclusion criteria will be allocated by computer randomized program that will be created by a statistician who is not otherwise involved in the trial and does not participate in analysis or interpretation of the results. The allocation sequence will be concealed from the principal investigator enrolling and assessing participants as each participant’s allocation which will be contained in sequentially numbered sealed and stapled opaque envelopes. All the eligible subjects will be allocated into G1, G2, and G3. Each group will recruit 30 subjects. The assessor will be blinded to the patient’s treatment group assignment at all time and will carry all the baseline, 4-week and 8-week evaluations.

Inclusion criteria were based on previous relevant studies (Itoh et al., 2008; Sánchez-Romero et al., 2018). Inclusion criteria were as follows: 1) >50 to 80 years old, 2) referred with a diagnosis of KOA (i.e., primary KOA fulfilling the American College of Rheumatology criteria developed by Altman et al. (1986) (at least three out of six) for clinical and radiographical diagnostic KOA 3) presenting with anterior and/or medial knee pain with 2-3 local tender spots, 4) crepitus, 5) functional limitations caused by KOA over a period of at least six months, 6) stiffness <30 minutes, 7) KL scale (Petersson et al.,1997) of grade I to grade III; and 8) able to read Chinese and communicate in Cantonese based on literacy.

Exclusion criteria, made with reference to previous studies (Itoh et al., 2008; Sánchez-Romero et al., 2018), were: 1) having knee pain less than 6 months; 2) having other musculoskeletal diseases associated with knee pain (e.g., referred pain from the low back or posterior or lateral knee pain or co-existing pain over the other limb); 2) suffering from acute inflammation, diffuse tenderness upon palpation test, bone marrow lesion, severe joint deformity with X-rays revealing a grade IV in the KL scale (Petersson et al., 1997), coagulation disorders, metabolic, or neuropathic arthropathies, immunosuppressed or systematic disease; 3) having severe concomitant illness that might affect the clinical outcomes of this study, 4) contraindications to DN including pregnancy, malignancy, fear of DN; 5) having recent treatment involving acupuncture or DN therapy or non-pharmacological intervention (e.g., physiotherapy) within a month prior to the start of the study; 6) inability to answer questionnaires and non-responsiveness towards the assessor; 7) having a wound or pressure sore or skin problems or skin allergy, including an allergy to iodine; and 8) having a history of injecting steroids.

Participants will be provided with written information sheets and verbal explanations. Written consent will be signed. They can withdraw from the study at any time. All the adverse effects, if any, will be recorded and reported in this study. The group allocation and data will be concealed in a password-protected computer file only accessible by the principal investigator.

Table 1: Participants flow chart:

Recruit from advertisements on social media and assessed by orthopedic surgeon for eligibility of KOA

Assessed for eligibility with inclusion and exclusion criteria

Obtaining written consent

Randomized with computer program by an independent statistician and the allocation sequence will be concealed with sealed and stapled opaque envelopes

Baseline assessment

G2

DN with placebo US-guide+

supervised exercise, exercise pamphlet and educational video

G1

DN + real US-guide+ routine supervised exercise and exercise pamphlet, educational video

G3

Control from waiting list without any treatment

Follow up immediate after the last session at the 4-week and 8-week follow-ups, and data analysis and post intervention analysis with mixed model ANOVA

1. Sample Size and rational for calculation

The effect size is calculated by using G-Power version 3.1.9.4 (Franz Faul, Universität Kiel, Germany)

With reference previous similar study of using acupuncture on KOA (Williamson et al., 2007), the effect size of VAS is set as 0.339 for the VAS, power as 0.8, level of statistical significance as 0.05. The number of subjects estimated for each group will be 26; total number of subjects was 78.

1. Method of data analysis:

Statistical analyses will be performed on the Intention to treat (ITT) population, including all randomized participants. Missing data will be replaced using multiple imputation (5 iterations). Intention to treat (ITT) analysis will be used in the data analysis. The data will be analyzed by using mixed model analysis of variance (ANOVA) (two-tailed) with ‘time’ as the within subjects factor (Baseline, 4 weeks and 8 weeks after treatment), whereas the ‘group’ factor will be analyzed for the between groups differences (G1, G2 and G3). The significant level will be set at 0.05 and bonferroni correction will be applied. Wilks’ Lambda will be used for statistic significance analysis. The assumption of sphericity will be tested by the Mauchly’s test and the assumption of normality will be tested by the Shapiro-Wilk test. Post hoc analysis by will be conducted if significant difference is found in the corresponding outcomes measures so as to identify the difference amongst the comparisons between groups. All statistical analysis will be conducted using SPSS version 23 (IMB, SPSS) and all analyses will be processed blind to identify of treatment groups by an independent research assistant.

1. Details of procedure of applying US-guided DN

After taking the baseline information and randomized into 3 groups (G1, G2 & G3). Subjects in G1 will receive needling and real US scanning, and routine exercise training and education of knee care for 4 weeks and once per week while subjects in G2 will receive DN with placebo US scanning, and routine exercise training and education of knee care for 4 weeks and once per week. Subjects in G3 will be measured all the outcomes at the baseline, at the 4-week and 8-week follow-ups. For G1, the US scanning procedure is done with the subject lies on a bed with pillow in a supine position, and with knee joint supported by a towel in an around 30 degrees flexed position (Appendix VI). For G1, the physiotherapist who received special training for US scanning procedure together with the DN intervention in the Hong Kong Polytechnic University will conduct the US scanning. An US machine with high frequency (4-12MHz) linear probe [Proble Model: L12-4 broadband linear array; US Machine: Philips Lumify US system [Koninklijke Philips N.V., Eindhoven, The Netherlands] is used to assess any sign of tendinopathy over the patello-femoral and medial patello-tibial compartments (Sengupta et al., 2006) with following characteristics: mucoid degenerative changes, heterogeneity or thickening of ligaments, osteophytes, meniscus tear, hyaline cartilage thinning in trochlear cartilage defects with reference to the Outcome Measures in Rheumatology (OMERACT) US Task Force on knee OA (Bruyn et al., 2014). To avoid anisotropy, there are two views that should be used included long axis and short axis to confirm pathology (Jacobson, 2018). With integration to detail physical examination, a physical diagnosis will be made. US-guided DN will be applied to the pathological structures e.g. tendinopathy over the patella-femoral and medial patella-tibial compartments. It is a tailor-made treatment and it will last for 15 minutes and the number of needling depending on the subject’s condition. The maximum of sterile needles with the size of Dongbang Acupuncture Needles at 0.3 x 40 mm [Dong Bang Acupuncture, Inc., Marsh Barton, Exeter, United Kingdom] will be seven or below (Chung & Binkley, 2010). The number of needles used will be recorded for analysis. The procedure was performed by the physiotherapist with sterile technique by disinfecting the US probe with a wet towel [Clinell Universal Wipes, GAMA Healthcare Ltd, Hemel Hempstead, Hertfordshire], covered it with a tegaderm [3M; St. Paul, MN, USA], and used Aquasonic sterile transmission gel [Aquasonic sterile transmission gel; Parker Laboratories, INC] for the transmission of US energy to the subject. The subject’s skin will be disinfected with Betadine antiseptic solution [Mundipharma Pharmaceuticals LTD., Nicosia, Cyprus] over the targeted DN sites. Then physiotherapist will use left hand to do the US scanning and right hand will apply the DN to the target location as planned. Based on clinical experience and the procedure of DN on KOA by previous studies (Cotchett et al., 2011; Tabatabaiee et al., 2019), good response has been observed with the following protocol: the needle will be slowly moving in and out of the target muscle or tendon with the goal of eliciting an appropriate response such as a local twitching response (LTR); sensation such as a dull ache, heaviness, distension, or a reproduction of the participant’s symptoms. Then the needling will be manipulated in and out slowly every 5 minutes for 5 times to reproduce the appropriate response, and the whole treatment time will be 15 minutes. After the treatment finished, sterile gauze [A.R. Medicom Inc. (Asia) Ltd, HKSAR, China] will be applied and pressed on the side of DN. Spot tapes sterile non-stick pad [Orison TM, HKSAR, China] will be used to cover the sites received DN. The case in-charged physiotherapist will state with the subject for the whole procedure to ensure safety.

For the G2, the subject will also be positioned in a supine position same as G1. Then the physiotherapist (same therapist as in G1) will wash hand and take sterile gloves, and then do the disinfection to the skin in the target DN sites. DN will be applied to the most painful sites. To blind the patients with a placebo US-guide, the physiotherapist will do the procedure of scanning as G1 but with US monitor’s screen, out-of-view from the G2 patient’s vantage point, was turned off to blind the subjects. Then the needling will be manipulated every 5 minutes and the whole treatment time will be 15 minutes. The whole procedure similar as mentioned in G1, but there is no US scanning. After the treatment finished, sterile gauze will be applied and pressed on the side of DN. The physiotherapist will state with the subject for the whole procedure to ensure safety.

For G3, the subjects received only standardized exercise therapy and educational materials related to the care of their knees. The exercise therapy was conducted under the supervision of a registered physiotherapist weekly for 4 sessions. The exercises focused on improving knee mobility, soft tissue flexibility, muscle strength, balance and proprioception. The participants were instructed to continue the exercises at home three times per week for another four weeks.

All the outcomes will be assessed by an independent research assistant.

Details of outcomes assessment

1. The Visual Analogue Scale (VAS) is commonly used in measuring musculoskeletal pain with a 100mm horizontal line anchored with labels on top of it (Appendix 1). The reliability of the VAS for disability is moderate to good (Boonstra et al., 2008) and VAS is the most reliable tool in assessing pain as compared with numerical rating scale (NRS) and verbal rating scale (VRS), with the smallest errors in the measurement of osteoarthritis knee pain (Alghadir et al., 2018). The minimum clinically important difference (MCID) was 19.9 mm (Katz et al., 2015; Tubach et al., 2005).
2. Knee injury and Osteoarthritis Outcome Score (KOOS)-short form is validated to be used in KOA patients (Lyman et al., 2016). KOOS is a valid, reliable and responsive outcomes measure, and it can improve the validity as compared with WOMAC (Roos et al., 2003). The Chinese translated version of KOOS is a reliable and valid instrument for OAK patients and showed fairly strong association with WOMAC (Cheung, Ngai and Ho., 2016). As WOMAC requires license and cost, KOOS is free to be used, KOOS already has been commonly used as a functional outcomes in most public hospitals in Hong Kong for KOA patients. The KOOS-short form involves three domains including pain, symptoms and quality-of-life (QoL). The score from ‘0’ to ‘4’ indicated none to severe and then the KOOS pain score can be calculated with the following formula:

KOOS-pain= 100 – (mean score of pain domain x 100)/4

The values of KOOS-subscales (symptoms and QoL) can be assessed by the above formula.

1. Evaluation of the Validity of placebo method

This study involves US-guide and stated clearly in the consent form and information sheet provided to very subjects. If one the study group does not provide US-guide, they may know that they are not allocated into the intervention group. To avoid the bias and potential placebo effect from US-guide, it is necessary to blind the subjects during the procedure of US-guide DN. All the subjects in G1 and G2 will receive either real-US-guide or placebo US-guide with US monitor’s screen, out-of-view from the G2 patient’s vantage point, was turned off to blind the subjects.

To ensure it, all the subjects in G1 and G2 will be asked whether they are receiving a real US-guide or a placebo US-guide DN. Is placebo US-guide really effective to service the purpose of blinding the subjects? Then the data will be collected for analysis by Chi-squared test for the nominal data with using SPSS version 23 (IMB, SPSS) and an alpha is set at 0.05.

**Benefits:**

The current study aims to investigate the effects of US-guided DN with exercise therapy on KOA patients using a comprehensive evaluation including pain level and functional ability. The findings to be obtained from this study will have a great potential to help the rehabilitation of patients with KOA in an efficient and safe manner. If US-guided DN can enhance the effectiveness of DN with exercise in improving pain and functional ability in elderly people with KOA, it potentially helps to reduce burden of total knee replacement operations to public hospitals and mortality rate. It may also release the stress of elderly patients onto the public healthcare system and to cope with the demands from the aging population in the coming future.

**Major ethical issues:**

DN or conventional acupuncture has been commonly used in patients with musculoskeletal problems. Patients may feel some dull arch feeling initially when the needling being puncture into the skin. Then patients usually will be able to adapt it. Some patients may have some unwell feeling or fainting or nausea shortly. It has been reported that the incident rate of these minor adverse events is 1.3 (0.9-1.7) per 1000 treatments; and there are no reports on serious adverse events, such as events requiring hospital admission, leading to permanent disability, or resulting in death (95% confident interval 0 to 1.1 per 10000 treatment) (MacPherson et al., 2001). If the subject feels any discomfort, the treatment will be stopped, and removal of needle immediately. The procedure will be done by a physiotherapist with accreditation in acupuncture and with experience in treating KOA by DN and clinical experience in out-patient unit for more than 10 years. The whole procedure of needling is done in supine position and the risk of fall can be minimized. Another potential risk of DN or conventional acupuncture is infection. The can be prevented by proper disinfection procedure and all the subjects’ needling points will be disinfected with the Betadine antiseptic solution [Mundipharma Pharmaceuticals LTD., Nicosia, Cyprus] and the head of US probe will be disinfected with sterile wet paper towel [Laboratories ANIOS, Lille-Hellemmes, France], and covered by a sterile 3M Tegaderm [3M, St. Paul, Minneapois, U.S.A.], and Aquagel sterile lubricating jelly [ECOLAB, Saint Paul, Minnesota, U.S.A.] as a transmission media for US scanning. All these needles being used in this study are sterile and disposable needles. The maximum of needles with the size of Dongbang Acupncture Needles at 0.3 x 40 mm [Dong Bang Acupuncture, Inc., Marsh Barton, Exeter, United Kingdom] will be seven or below. All the needles will be used once only. The risk of infection can further minimize with the exclusion criteria of any immunosuppressed diseases or under steroid treatment. From previous literatures, there were only 3 patients out of 962 patients (0.31%) who reported hypodermal bleeding and (2.1%) complained of gastrointestinal problems including nausea, stomachache, dyspepsia, and headache [Qin et al., 2015]. It may rarely happen in some cases and it will recover after rest. The procedure will be clearly explained to the subjects and the procedure will be terminated if subjects find it is too uncomfortable. Currently, the World Health Organization (2019) recommends acupuncture for over 100 diseases inducing knee pain. They encourage and support countries to identify safe and effective remedies and practices for use in public and private health services. The whole procedure of needling will be conducted in a clean working environment. The physiotherapist should clean hands and wearing sterile gloves, use disinfectant [Betadine antiseptic solution] to prepare of the needling sites and all sterile needles will be applied with an aseptic technique; and finally careful management and disposal of used needles and swabs. After the treatment finished, sterile gauze [Safe Gauze, A.R. Medicom Inc. (Asia) Ltd., Hong Kong] will be applied and pressed on the side of DN. Spot tapes sterile non-stick pad [Orison ^TM^, Korea] will be used to cover the sites received needling treatment. The risk for septic complications and other drug related side effects related to musculoskeletal US-guided maneuvers is very low (Micu et al., 2014). Continuous monitoring the subjects’ feeling during the whole procedure, if there is any discomfort, proper management will be provided whenever necessary. It can ensure the safety and comfort and the rights of all subjects.

Appendix I

Visual analogue scale (VAS) charts (Alghadir et al., 2018; Boonstra et al., 2008).


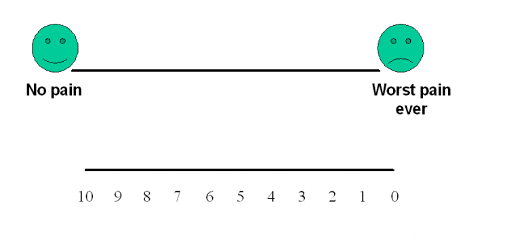


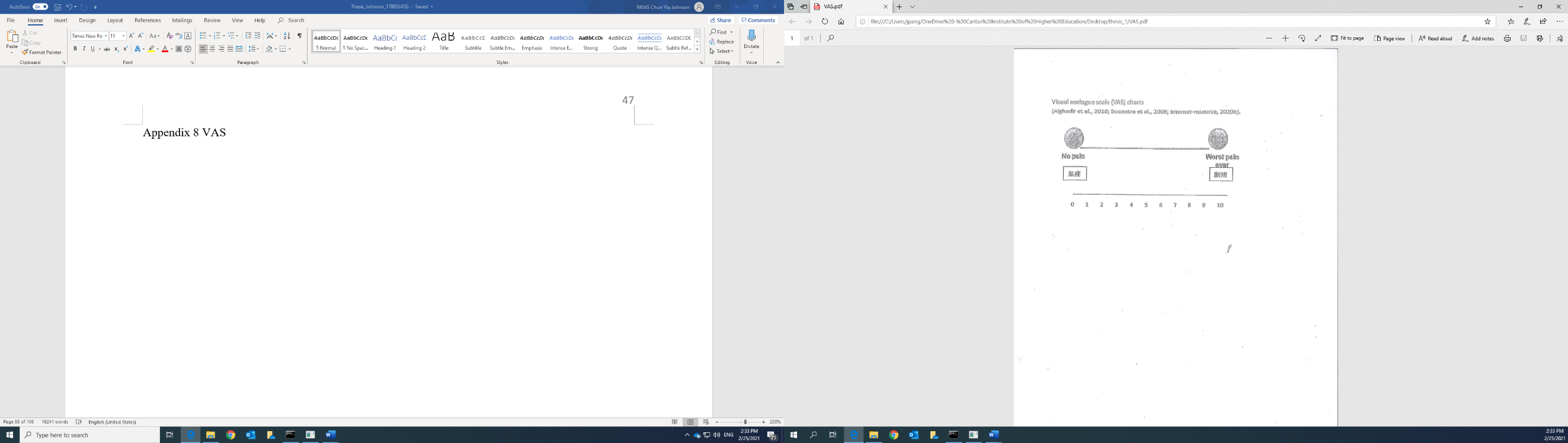


無痛

劇痛

Appendix II

Chinese version KOOS questionnaire (Roos et al., 2003; Cheung, Ngai and Ho., 2016)


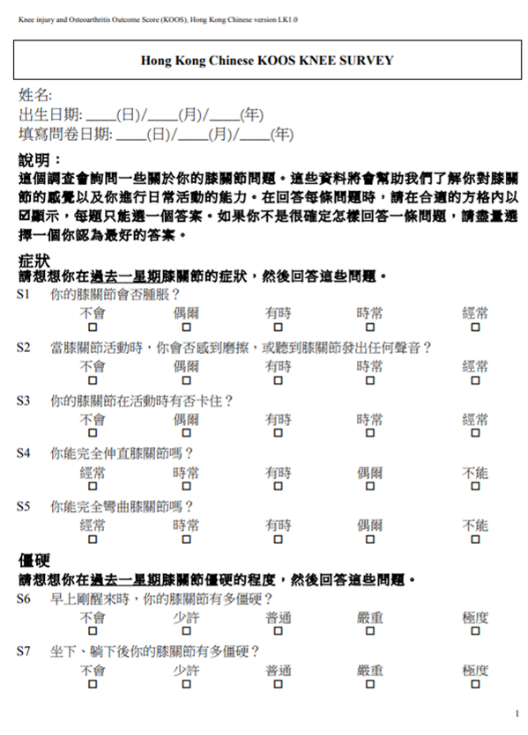


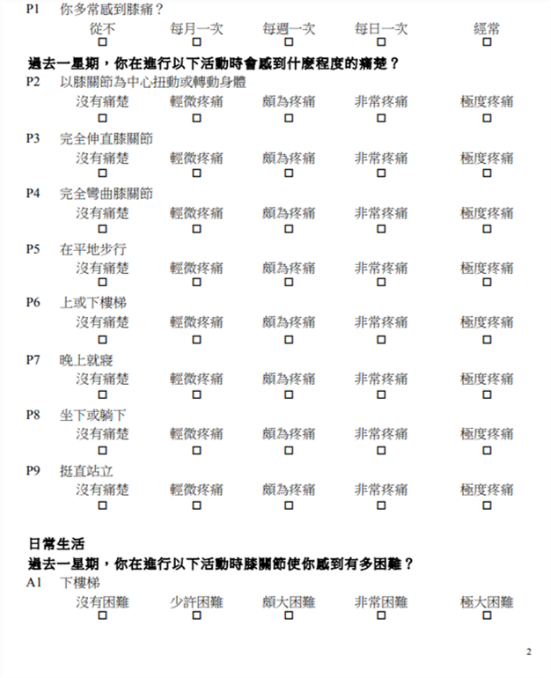


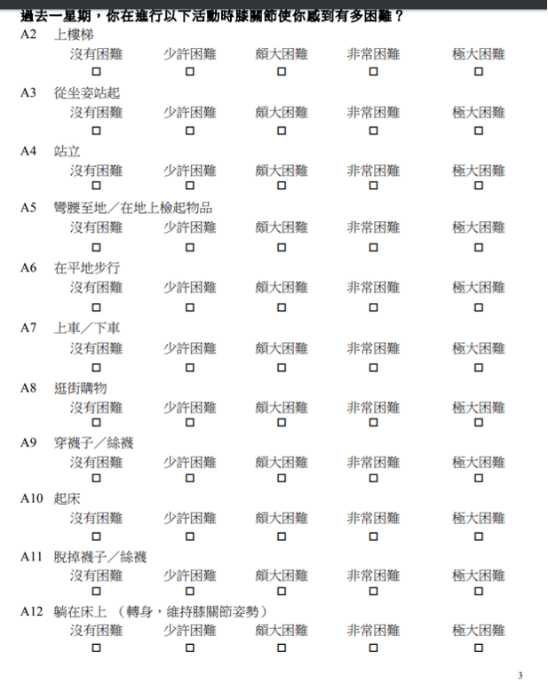


Appendix III

Kellgren and Lawrence system (Petersson et al., 1997)

Grade 0: no radiographic features of OA are present

Grade I: doubtful joint space narrowing (JSN) and possible osteophytic lipping

Grade II: definite osteophytes and possible JSN on anteroposterior weight-bearing radiograph

Grade III: multiple osteophytes, definite JSN, sclerosis, possible bony deformity

Grade IV: large osteophytes, marked JSN, severe sclerosis and definite bony deformity

Appendix IV

Data collection form

| Case identity | Date of assessment | Age | Gender | | | BMI | | Unilateral/Bilateral knee pain | | Duration of knee pain | Any injury |
| --- | --- | --- | --- | --- | --- | --- | --- | --- | --- | --- | --- |
|  |  |  | M / F | | | Height: M  Weight: Kg | | R ______  L ______  Both ___ | | ___months | (Y / N) |
| Severity of pain (VAS) | KOOS-Pain score | KOOS-  symptoms  score | KOOS_ADL score | | | Remarks (any any discomfort or side effects): | | | | | |
| _____ mm | ______/36 | ______/28 | ______/68 | | |
|  |  |  |  | | |
| Number of needle used  ( ) | Areas applied dry needling or acupuncture  Oblique/perpendicular insertion: / | | | | |  | Nausea/faint/ hypodermal bleeding others:  _______________________________ | | | | |
| Compliance of exercises: |  | | | | XR: KL scale: Grade 0 / I / II /III /IV | | | | | | |
| Tablets of medication |  | | | Number of tender spots: | | | | | Depth of insertion (mm): | | |
| Ultrasound findings | Patient code: ___________  Real US-dry needling: Y/N  Findings: __Power Doppler of blood flow increased/decreased (neovascularity +/-)  Mucoid degeneration/increased thickness/hypoechogenecicity/hyperechogenecity  __________________________________________________________________________________  __________________________________________________________________________________  __________________________________________________________________________________ | | | | | | | | | | |
| Location of dry needling | 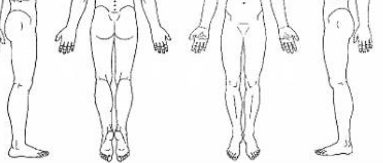  L medial  R medial | | | | | | | | | | |

Key: Q= quadriceps strength, H= hamstrings strength, ROM=range of motion, R= right and L= left, M=male, F=female, Y= yes and N= no, MMT: manual muscle testing

Scales: mm= minimeter, N=newton, ^0^ is degree of angle

Appendix V

US scanning procedure (Jacobson 2018).

Part 1. Anterior patella-femoral compartment:

Patello-femoral compartment (Figure 1 of Appendix VI) (Sengupta et al. 2006): the structures will be evaluated from anterior part of the knee joint including the quadriceps muscles, quadriceps tendon, the patella, the patella tendon, the patella retinaculum, the suprapatellar recess, the medial and lateral recesses (Table 1 of Appendix VI).

1. Examination will be begun in the sagittal plane 2 cm proximal to the base of patella (Figures 2A and 2B of Appendix VI). Slight flexion of the knee in 30^0^ with a roll of towel behind the knee that is often helpful because this position straightens and tenses the extensor mechanism to reduce tendon anisotropy.
2. The quadriceps tendon is then evaluated in short axis (Figures 3A and 3B of Appendix VI)
3. Then the US probe is turned back to sagittal and milks the suprapatellar recess by shifting the joint fluid from other distal part of the knee joint to see any abnormal joint effusion.
4. The transducer is then moved inferiorly below the patella in the sagittal plane to exam the patellar tendon whether it is in a hyperechoic, fibrillar, and uniform pattern (Figures 4A and 4B of Appendix VI).
5. The Hoffa infrapatellar fat pad appears slightly hyperechoic and underneath to the patellar tendon. The region around the distal patellar tendon is also evaluated for superficial and deep infrapatellar bursal fluid; minimal fluid is considered to be normal pattern (Figures 5A and 5B of Appendix VI).
6. The transducer is then moved to both the medial and lateral margins of the patella in the transverse plane to see the thin hyperechoic patellar retinaculum. With distention of the medial and lateral recesses by milk them with joint fluid from the surrounding structure that are continuous examination with the suprapatellar recess, which is more apparent when the knee is completely extended. In addition, the medial patellofemoral ligament (MPFL) and patello-tibial ligament (MPTL) will also be visualized (Figures 6A and 6B of Appendix VI). The medial patellofemoral ligament is located from the medial femoral epicondyle and adductor tubercle running obliquely to insert to the upper 2/3 medial border of the patella (Figures 6A and 6B of Appendix VI). The patella-tibial ligament is origin from medial tibial tubercle and run slightly obliquely to insert to the medial lower1/3 of the medial patella (Figures 7A and 7B of Appendix VI).
7. Finally, with the knee in flexion, the hypoechoic hyaline cartilage that covers the trochlea of the anterior femur can be appreciated in the transverse plane superior to the patella. A uniform thin layer of hypoechoic hyaline cartilage covering the anterior and central aspects of the femoral condyles can be seen in the parasagittal plane in non-pathological subjects (Figures 8A and 8B of Appendix VI).

Part 2: medial tibio-femoral compartment (Sengupta et al. 2006):

1. For medial knee evaluation, the patient remains supine and slightly rotates the hip externally to gain access to the medial knee structures. The structures of interest include the medial collateral ligament, the anterior horn and medial part of the body of the medial meniscus, and the pes anserinus. To begin, the transducer is placed in the coronal plane along the medial joint line, which is identified by the bone contours of the femoral condyle and the proximal tibia (Figures 9A and 9B of Appendix VI). The medial collateral ligament appears as a thick hyperechoic and fibrillar superficial layer that is easily identified in long axis; it extends proximally from the medial femoral condyle, and distally to the slightly antetior proximal tibial metaphysis.
2. With turning of the transducer short axis to the medial collateral ligament, the anteroposterior extent of this structure can be appreciated (Figures 10A and 10B of Appendix VI).
3. Then the transducer will be turned back to the long axis to the tibial collateral ligament, thinner hyperechoic deep layers of the medial collateral ligament, also called the meniscofemoral and meniscotibial ligaments, are identified from the meniscus to the femur and tibia, respectively (Figure 9B of Appendix VI). The fibrocartilage meniscus is located as a triangular hyperechoic structure between the femur and the tibia. The transducer is then moved anteriorly from the coronal plane in the oblique-sagittal plane to examine the anterior horn of the medial meniscus.
4. The transducer is then moved distally below the joint line along the tibial collateral ligament and slightly anterior to its attachment on the tibia, around 4 cm below the joint line (Figures 11A and 11B of Appendix VI). Here, the pes anserinus can be seen as three hyperechoic tendons superficial to the tibial collateral ligament that converge onto the tibia and in a sequence of Sartorius, Gracilis and Semitendinosus tendons.

**Appendix VI**

Knee sonographic examination-checklist (Jacobson 2018).

| 1. Anterior and medial patellofemoral compartment |
| --- |
| 1. The most common anatomical locations of pain in OAK patients   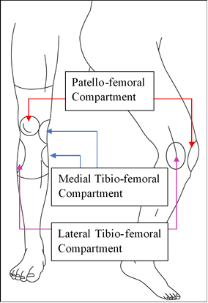  **Appendix VI Figure 1.** The most common anatomical locations of pain in OAK patients (Sengupta et al. 2006). |
| **Appendix VI Figure 2A**. shows the position of anterior knee US scanning for the Sagittal view and 2B shows the quadriceps tendon (black arrowheads), quadriceps fat pad (Q), prefemoral fat pad (PF), and collapsed joint recess (white arrow), F=femur and P=patella (Jacobson 2018).  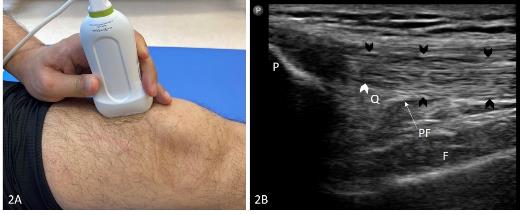 |
| **Appendix VI Figure 3A and 3B.** Short axis of quadriceps femoris (Figure 3A) and the quadriceps tendon (arrowheads) (Figure 3A).  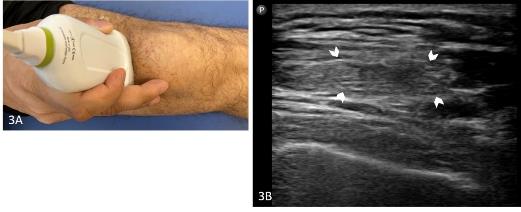 |
| **Appendix VI Figure 4A and 4B.** US scanning to patella tendon in sagittal view (Figure 4A) and the patella tendon (arrowheads) and Hoffa fat pad (H). P=patella and T=tibia in (Figure 4B).  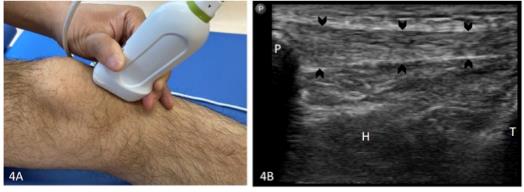 |
| **Appendix VI Figure 5A.** US scanning to patella tendon in transverse view (Figure 5A) and the patella tendon (arrowheads) and Hoffa fat pad (H) in Figure 5B.  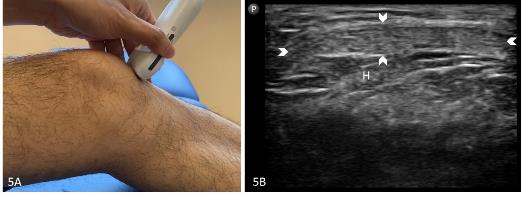 |
| **Appendix VI Figure 6A**. US scanning to patellofemoral tendon in longitudinal view (Figure 6A). The medial knee joint recess (white arrow) and the arrowheads show the patellofemoral ligament (Figure 6B) and position of patellofemoral ligament in medial knee (Figure 6C). F=femoral condyle, P=patella.  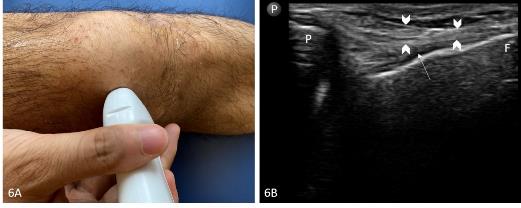  **Appendix VI Figure 6C.** The location of the medial patellotibial ligament (MPTL), medial patellofemoral ligament (MPFL) and it runs obliquely and transverse origin from the femoral epicondyle and adductor tubercle. Around the vastus medialis oblique muscle (VMO), the medial collateral ligament (MCL), and the adductor magnus tendon (AMT) are outlined with dotted lines (Jacobson 2018).  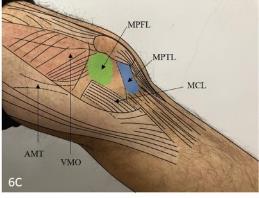 |
| **Appendix VI Figure 7A**. US scanning to patellotibial tendon in longitudinal view (Figure 7A) and position of patellotibial ligament in medial knee (Figure 6C). The arrowheads show the patellotibial ligament (Figure 7B). T=tibial condyle, P=patella.  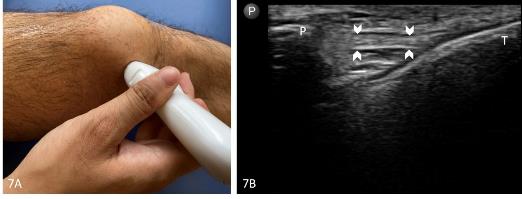 |
| **Appendix VI Figure 8A.** Trochlear and femoral condyle cartilage. Figure 8A shows hypoechoic hyaline cartilage (arrowheads). LC=lateral femoral condyle, MC=medial femoral condyle, P=patella.  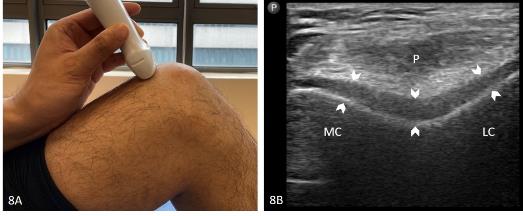 |
| **Appendix VI Figure 9A.** Medial knee sonography evaluation in coronal plane (Figure 9A). Coronal imaging at the medial joint line is shown in (Figure 9B) the superficial (arrows) and deep (arrowheads) layers of the medial collateral ligament, and medial meniscus (white arrow). F=femur, T=tibia.  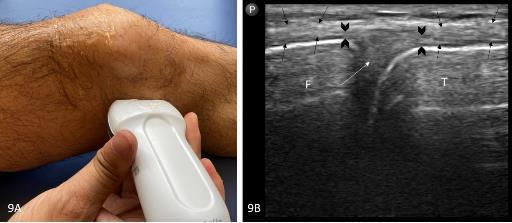 |
| **Appendix VI Figure 10A**. Sonography examination of the medial collateral ligament in transverse plane (Figure 10A). Transverse imaging shows the medial collateral ligament (arrowheads). F=femur.  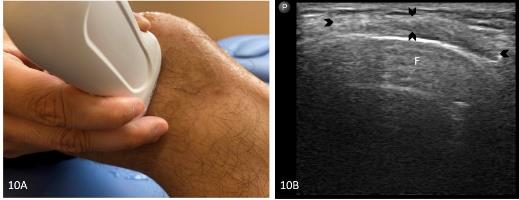 |
| **Appendix VI Figure 11A.** Distal medial collateral ligament and pes anserinus. Coronal imaging distal to knee joint shows (Figure 11A), the superficial layer of the medial collateral ligament (arrowheads) and the tendons of the pes anserinus (arrows) (T=tibial metaphysis). Sonographic imaging in long axis shows the semitendinosus proximal to pes anserinus (arrowheads).  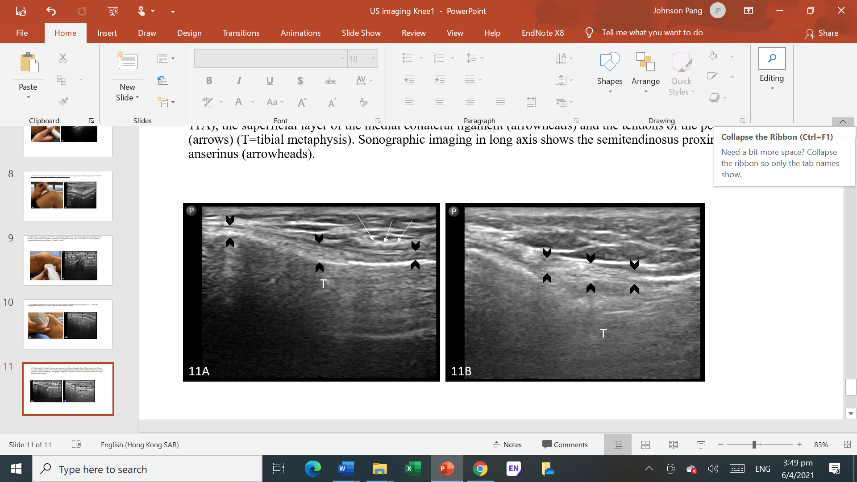 |

**Appendix VII**

Supervised exercise pamphlet


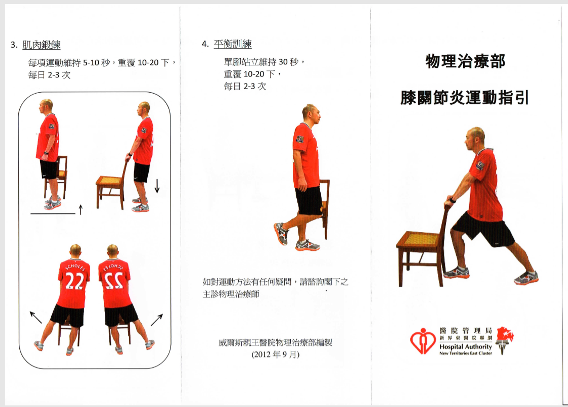


Reference

Alghadir, A. H., S. Anwer, A. Iqbal, and Z. A. Iqbal. 2018. "Test-Retest Reliability, Validity, and Minimum Detectable Change of Visual Analog, Numerical Rating, and Verbal Rating Scales for Measurement of Osteoarthritic Knee Pain." *J Pain Res* 11: 851-856. <https://dx.doi.org/10.2147/jpr.S158847>.

Altman, R., Asch, E., Bloch, D., Bole, G., Borenstein, D., Brandt, K., . . . Wolfe, F. (1986). Development of criteria for the classification and reporting of osteoarthritis: Classification of osteoarthritis of the knee. *Arthritis and rheumatism, 29*(8), 1039-1049. doi:10.1002/art.1780290816

Andrianakos, A., Trontzas, P., Christoyannis, F., Dantis, P., Voudouris, C., Georgountzos, A., . . . Kontoyanni, A. (2003). Prevalence of rheumatic diseases in Greece: a cross-sectional population based epidemiological study. The ESORDIG Study. *J Rheumatol, 30*(7), 1589-1601.

Boonstra, M. Anne, R. Henrica Schiphorst Preuper, F. Michiel Reneman, B. Jitze Posthumus, and E. Roy Stewart. 2008. "Reliability and Validity of the Visual Analogue Scale for Disability in Patients with Chronic Musculoskeletal Pain." *International Journal of Rehabilitation Research* 31, no. 2: 165-169. <https://dx.doi.org/10.1097/MRR.0b013e3282fc0f93>.

Brandt, K. D., Heilman, D. K., Slemenda, C., Katz, B. P., Mazzuca, S., Braunstein, E. M., & Byrd, D. (2000). A comparison of lower extremity muscle strength, obesity, and depression scores in elderly subjects with knee pain with and without radiographic evidence of knee osteoarthritis. *J Rheumatol, 27*(8), 1937-1946.

Bruyn, G. A., E. Naredo, N. Damjanov, A. Bachta, P. Baudoin, H. B. Hammer, F. B. Lamers-Karnebeek, et al. 2016. "An Omeract Reliability Exercise of Inflammatory and Structural Abnormalities in Patients with Knee Osteoarthritis Using Ultrasound Assessment." *Ann Rheum Dis* 75, no. 5 (May): 842-6. <https://dx.doi.org/10.1136/annrheumdis-2014-206774>.

Cheung, R.T., S. P. Ngai, and K. K. Ho. 2016. "Chinese Adaptation and Validation of the Knee Injury and Osteoarthritis Outcome Score (Koos) in Patients with Knee Osteoarthritis." *Rheumatol Int* 36, no. 10 (Oct): 1449-54. <https://dx.doi.org/10.1007/s00296-016-3539-7>.

Chung, G., Binkley, H. 2010. "Acupuncture and Knee Conditions. Athletic Training & Sports Health Care." *The Journal for the Practicing Clinician* 6, no. 2: 278-286

Colberg, R. E., and R. G. Henderson. 2017. "Diagnosis and Treatment of Gouty Tophi in the Patellar Tendon Using Ultrasound-Guided Needle Barbotage: A Case Presentation." *Pm r* 9, no. 9 (Sep): 938-942. <https://dx.doi.org/10.1016/j.pmrj.2016.12.013>.

Cotchett, Matthew P., Karl B. Landorf, Shannon E. Munteanu, and Anita Raspovic. 2011. "Effectiveness of Trigger Point Dry Needling for Plantar Heel Pain: Study Protocol for a Randomised Controlled Trial." *Journal of foot and ankle research* 4, no. 1: 5-5. <https://dx.doi.org/10.1186/1757-1146-4-5>.

Cowan, S. M., Bennell, K. L., Hodges, P. W., Crossley, K. M., & McConnell, J. (2001). Delayed onset of electromyographic activity of vastus medialis obliquus relative to vastus lateralis in subjects with patellofemoral pain syndrome. *Archives of Physical Medicine and Rehabilitation, 82*(2), 183-189. doi:10.1053/apmr.2001.19022

Daftary, Aditya, and Alpana Karnik. 2015. "Perspectives in Ultrasound-Guided Musculoskeletal Interventions." *The Indian Journal of Radiology and Imaging* 25, no. 3: 246-260. <https://dx.doi.org/10.4103/0971-3026.161445>.

Davidson, J., and S. Jayaraman. 2011. "Guided Interventions in Musculoskeletal Ultrasound: What's the Evidence?" *Clin Radiol* 66, no. 2 (Feb): 140-52. <https://dx.doi.org/10.1016/j.crad.2010.09.006>.

Fabbro, Emanuele, Giulio Ferrero, Davide Orlandi, Chiara Martini, Francesca Nosenzo, Giovanni Serafini, Enzo Silvestri, and Luca Maria Sconfienza. 2012. "Rotator Cuff Ultrasound-Guided Procedures: Technical and Outcome Improvements." *Imaging in medicine* 4, no. 6: 649-656. <https://dx.doi.org/10.2217/iim.12.57>.

Felson, D. T., Lawrence, R. C., Hochberg, M. C., McAlindon, T., Dieppe, P. A., Minor, M. A., . . . Goldberg, V. (2000). Osteoarthritis: new insights. Part 2: treatment approaches. *Annals of internal medicine, 133*(9), 726-737. doi:10.7326/0003-4819-133-9-200011070-00015

Granvir, S. D., Zambare, B.R., Naikwade, D.B. . (2015). Effects of open chain exercises on muscle strength and function in elderly patients with knee Osteoarthritis. . *International Archives of Integrated Medicine, 2*(4), 5-10.

Harkey, M. S., Price, L. L., McAlindon, T. E., Davis, J. E., Stout, A. C., Lu, B., . . . Driban, J. B. (2019). Association Between Declining Walking Speed and Increasing Bone Marrow Lesion and Effusion Volume in Individuals with Accelerated Knee Osteoarthritis. *Arthritis Care Res (Hoboken), 71*(2), 259-270. doi:10.1002/acr.23613

<https://dx.doi.org/10.1002/mus.26671>.

Itoh, K., Hirota, S., Katsumi, Y., Ochi, H., & Kitakoji, H. (2008). Trigger point acupuncture for treatment of knee osteoarthritis – a preliminary RCT for a pragmatic trial. *Acupunct Med, 26*(1), 17-26. doi:10.1136/aim.26.1.17

Jacobson, J. 2018. *Fundamentals of Musculoskeletal Ultrasound (Third Ed.)*. Fundamentals of radiology. Philadelphia: PA: Elsevier.

James, S, K Ali, C Pocock, C Robertson, J Walter, J. Bell, . . ., and C Bradshaw. 2007. "Ultrasound Guided Dry Needling and Autologous Blood Injection for Patellar Tendinosis." *British Journal of Sports Medicine* 41, no. 8: 518-21; discussion 522.

Katz NP, Paillard FC, Ekman E. Determining the clinical importance of treatment benefits for interventions for painful orthopedic conditions. Journal of orthopaedic surgery and research. 2015;10:24

Lindseth, F., Lango, T., Selbekk, T., Hansen, R.,…Hernes, T. A. N. 2013. "Ultrasound-Based Guidance and Therapydoi: 10.5772/55884. Retrieved on 27 February 2020: [Https://Www.Intechopen.Com/Books/Advancements-and-Breakthroughs-in-Ultrasound-Imaging/Ultrasound-Based-Guidance-and-Therapy](https://Www.Intechopen.Com/Books/Advancements-and-Breakthroughs-in-Ultrasound-Imaging/Ultrasound-Based-Guidance-and-Therapy). . ."

Lungu, Eugen, Philippe Grondin, Patrice Tétreault, François Desmeules, Guy Cloutier, Manon Choinière, and Nathalie Bureau. 2018. "Ultrasound-Guided Tendon Fenestration Versus Open-Release Surgery for the Treatment of Chronic Lateral Epicondylosis of the Elbow: Protocol for a Prospective, Randomised, Single Blinded Study." *BMJ Open* 8, no. 6. <https://dx.doi.org/10.1136/bmjopen-2017-021373>.

Lyman, S., Lee, Y.-Y., Franklin, P. D., Li, W., Cross, M. B., & Padgett, D. E. (2016). Validation of the KOOS, JR: A Short-form Knee Arthroplasty Outcomes Survey. *Clinical Orthopaedics and Related Research®*, *474*(6), 1461-1471. <https://doi.org/10.1007/s11999-016-4719-1>

MacPherson, H., Thomas, K., Walters, S., & Fitter, M. (2001). The York acupuncture safety study: prospective survey of 34 000 treatments by traditional acupuncturists. *BMJ*, *323*(7311), 486. <https://doi.org/10.1136/bmj.323.7311.486>

McNally, Eugene G. 2011. "The Development and Clinical Applications of Musculoskeletal Ultrasound." *Skeletal Radiol* 40, no. 9: 1223-1231. <https://dx.doi.org/10.1007/s00256-011-1220-5>

Micu, M. C., Vlad, V. M., Bolboacă, S. D., Cârlig, M., Bodizs, G. I., Duţu, A. G., & Fodor, D. (2014). Musculoskeletal ultrasound guided manoeuvres - a security profile. Med Ultrason, 16(3), 214-221. https://doi.org/10.11152/mu.2013.2066.163.mcm1

Naredo, E., Cabero, F., Palop, M. J., Collado, P., Cruz, A., & Crespo, M. (2005). Ultrasonographic findings in knee osteoarthritis: A comparative study with clinical and radiographic assessment. *Osteoarthritis and Cartilage, 13*(7), 568-574. doi:10.1016/j.joca.2005.02.008

Petersson, I. F., Boegård, T., Saxne, T., Silman, A. J., & Svensson, B. (1997). Radiographic osteoarthritis of the knee classified by the Ahlbäck and Kellgren & Lawrence systems for the tibiofemoral joint in people aged 35–54 years with chronic knee pain. *Ann Rheum Dis, 56*(8), 493-496. doi:10.1136/ard.56.8.493

Qin, Z., Liu, X., Wu, J., Zhai, Y., & Liu, Z. (2015). Effectiveness of Acupuncture for Treating Sciatica: A Systematic Review and Meta-Analysis. *Evidence-Based Complementary and Alternative Medicine*, *2015*, 425108. <https://doi.org/10.1155/2015/425108>

Rathwa, A., Prajapati, N., & Deepak, A. N. (2019). To compare the effectiveness of proprioceptive circuit exercises versus open kinematics chain exercises on pain and improve muscle strength and physical function in osteoarthritis knee patients. *Ind. Jour. of Physioth. and Occupat. Therapy - An Inter. Jour., 13*(1), 88. doi:10.5958/0973-5674.2019.00017.0

Roos, E. M., & Toksvig-Larsen, S. (2003). Knee injury and Osteoarthritis Outcome Score (KOOS) – validation and comparison to the WOMAC in total knee replacement. *Health and Quality of Life Outcomes, 1*(1), 17-17. doi:10.1186/1477-7525-1-17

Sadatsuki, R., Ishijima, M., Kaneko, H., Liu, L., Futami, I., Hada, S., . . . Kaneko, K. (2019). Bone marrow lesion is associated with disability for activities of daily living in patients with early stage knee osteoarthritis. *J Bone Miner Metab, 37*(3), 529-536. doi:10.1007/s00774-018-0950-z

Samuel, A. J., & Kanimozhi, D. (2019). Outcome measures used in patient with knee osteoarthritis: With special importance on functional outcome measures. *International journal of health sciences*, *13*(1), 52.

Sánchez-Romero, E. A., Pecos-Martín, D., Calvo-Lobo, C., Ochoa-Sáez, V., Burgos-Caballero, V., & Fernández-Carnero, J. (2018). Effects of dry needling in an exercise program for older adults with knee osteoarthritis: A pilot clinical trial. *Medicine (Baltimore), 97*(26), e11255-e11255. doi:10.1097/MD.0000000000011255

Sengupta, M., Y. Q. Zhang, J. B. Niu, A. Guermazi, M. Grigorian, D. Gale, D. T. Felson, and D. J. Hunter. 2006. "High Signal in Knee Osteophytes Is Not Associated with Knee Pain." *Osteoarthritis and Cartilage* 14, no. 5: 413-417. <https://dx.doi.org/10.1016/j.joca.2005.11.012>.

Steultjens, M. P. M., Dekker, J., van Baar, M. E., Oostendorp, R. A. B., & Bijlsma, J. W. J. (2000). Range of joint motion and disability in patients with osteoarthritis of the knee or hip. *Rheumatology, 39*(9), 955-961. doi:10.1093/rheumatology/39.9.955

Tabatabaiee, Abbas, Ismail Ebrahimi Takamjani, Javad Sarrafzadeh, Reza Salehi, and Maryam Ahmadi. 2019. "Ultrasound‐Guided Dry Needling Decreases Pain in Patients with Piriformis Syndrome." *Muscle & Nerve* 60, no. 5: 558-565.

Tubach F, Ravaud P, Baron G, Falissard B, Logeart I, Bellamy N, et al. Evaluation of clinically relevant changes in patient reported outcomes in knee and hip osteoarthritis: the minimal clinically important improvement. Ann Rheum Dis. 2005;64(1):29-33.

Vaishya, Raju, Godfrey B. Pariyo, Amit Kumar Agarwal, and Vipul Vijay. 2016. "Non-Operative Management of Osteoarthritis of the Knee Joint." *Journal of Clinical Orthopaedics and Trauma* 7, no. 3: 170-176. <https://dx.doi.org/10.1016/j.jcot.2016.05.005>.

WHO. (2018). World Health Organization: Ageing and health. *Retrieved in the following website on 12 January 2020.* [*https://www.who.int/news-room/fact-sheets/detail/ageing-and-health*](https://www.who.int/news-room/fact-sheets/detail/ageing-and-health)

WHO. (2019). *The World Health Organization recommends acupuncture for over 100 conditions.* Retrieved in the following website on 13 January 2020: <https://holistic-health.org.uk/world-health-organisation-recommends-acupuncture-100-conditions/>

Williamson, L., M. R. Wyatt, K. Yein, and J. T. K. Melton. 2007. "Severe Knee Osteoarthritis: A Randomized Controlled Trial of Acupuncture, Physiotherapy (Supervised Exercise) and Standard Management for Patients Awaiting Knee Replacement." *Rheumatology* 46, no. 9: 1445-1449. <https://dx.doi.org/10.1093/rheumatology/kem119>.
