# Supplementary material for: Ultrasound-guided dry needling versus traditional dry needling for patients with knee osteoarthritis: A double-blind randomized controlled trial
Source: PLoS One. 2022 Sep 30;17(9):e0274990. doi: 10.1371/journal.pone.0274990 (PMC9524650; doi:10.1371/journal.pone.0274990)
Supplement: S1 Table — (PDF) [file pone.0274990.s005.pdf]

S1 Table. The raw data for the sonographic examination and painful spots during palpation in physical examination for the selection of sites for US-guided DN.

Subjects	Mucoid changes	Hypo-echogenicity	Hyper-echogenicity	Pain at MPFL	Pain at MPTL	Pain at MCL
1	●				●	
2	●			●		
3	●			●	●	
4	●			●	●	
5	●			●		
6	●				●	●
7	●			●	●	
8	●					●
9	●					●
10	●				●	●
11	●				●	●
12	●					●
13	●					●
14	●				●	
15	●					●
16	●			●	●	
17	●				●	●
18	●				●	●
19	●			●		
20			●	●	●	
21	●			●		
22		●		●		
Subjects	Mucoid changes	Hypo-echogenicity	Hyper-echogenicity	Pain at MPFL	Pain at MPTL	Pain at MCL

23			●	●		
24		●		●	●	
25		●			●	●
26	●			●	●	
27	●			●	●	
28	●			●		
29			●	●		
30			●	●		
31		●		●		
32	●			●		
33	●			●	●	
34	●			●	●	
35	●			●		
36	●				●	●
37	●			●		
38	●			●	●	
39	●			●	●	
40	●			●		
41		●		●		
42			●	●	●	
43	●				●	●
44	●				●	●

Subjects	Mucoid changes	Hypo-echogenicity	Hyper-echogenicity	Pain at MPFL	Pain at MPTL	Pain at MCL
45	●			●		

46	●	●	●	
47	●	●		
48	●		●	
49	●			●
50	●	●		
51	●	●	●	
52	●		●	
53	●	●	●	
54	●			●
55	●			●
56	●	●		●
57	●		●	●
58	●	●		
59	●		●	
60	●	●		

MPFL=medial patellofemoral ligament, MPTL=medial Patellotibial ligament, MCL=medial collateral ligament
