# Supplementary material for: Ultrasound-guided dry needling versus traditional dry needling for patients with knee osteoarthritis: A double-blind randomized controlled trial
Source: PLoS One. 2022 Sep 30;17(9):e0274990. doi: 10.1371/journal.pone.0274990 (PMC9524650; doi:10.1371/journal.pone.0274990)
Supplement: S2 Table — (PDF) [file pone.0274990.s006.pdf]

S2 Table. Number of tender spots and number of needles used.

The statistical analysis of tender spots and number of needles used for total four sessions.

	G1	G2	P-value
Number of tender spots	2.73±1.01	2.60±0.72	P=0.560
Number of needles used	12.97±4.49	12.31±3.38	P=0.546

The mean of tender spots in G1 and G2 was not significantly different (P=0.560) and the mean of needles used was also not significantly different (P=0.546).
