# Supplementary material for: Ultrasound-guided dry needling versus traditional dry needling for patients with knee osteoarthritis: A double-blind randomized controlled trial
Source: PLoS One. 2022 Sep 30;17(9):e0274990. doi: 10.1371/journal.pone.0274990 (PMC9524650; doi:10.1371/journal.pone.0274990)
Supplement: S3 Table — (PDF) [file pone.0274990.s007.pdf]

S3 Table. Sonographic findings and distribution of tender spots for US-guided DN (G1) and placebo US-guided DN (G2).

	Number of subject (G1)	Number of subject (G2)
Hypoechogenicity	2	3
Hyperechogenicity	4	1
Mucoid degeneration	24	26
MPFL	17	19
MPTL	16	15
MCL	11	7

MPFL=medial patellofemoral ligament, MPTL=medial Patellotibial ligament, MCL=medial collateral ligament
