# Supplementary material for: Ultrasound-guided dry needling versus traditional dry needling for patients with knee osteoarthritis: A double-blind randomized controlled trial
Source: PLoS One. 2022 Sep 30;17(9):e0274990. doi: 10.1371/journal.pone.0274990 (PMC9524650; doi:10.1371/journal.pone.0274990)
Supplement: S4 Table — (PDF) [file pone.0274990.s008.pdf]

S4 Table. Exercise compliance for the three groups at different time points.

	4-week (*Mean±SD*)	8-week (*Mean±SD*)
G1 (hours/week)	2.68±1.77	2.42±1.88
G2 (hours/week)	2.36±2.73	2.11±2.17
G3 (hours/week)	1.58±1.08	1.56±1.11

G1=Real US-guided DN with exercise therapy, G2=placebo US-guided DN with exercise therapy, and G3= exercise therapy solely
