# Supplementary material for: Ultrasound-guided dry needling versus traditional dry needling for patients with knee osteoarthritis: A double-blind randomized controlled trial
Source: PLoS One. 2022 Sep 30;17(9):e0274990. doi: 10.1371/journal.pone.0274990 (PMC9524650; doi:10.1371/journal.pone.0274990)
Supplement: S5 Table — (PDF) [file pone.0274990.s009.pdf]

S5 Table. The mean of pharmacological intervention for the three groups at different time points.

	Baseline *(Mean±SD)*	4-week *(Mean±SD)*	8-week *(Mean±SD)*
G1 (N/month)	2.76±10.66	0.69±3.71	0.14±0.74
G2 (N/month)	1.04±2.75	0.23±0.86	0.31±0.93
G3 (N/month)	2.34±11.19	2.28±11.16	0.14±0.74

G1=Real US-guided DN with exercise therapy, G2=placebo US-guided DN with exercise therapy, and G3= exercise therapy solely
